# TAS1553, a small molecule subunit interaction inhibitor of ribonucleotide reductase, exhibits antitumor activity by causing DNA replication stress

**DOI:** 10.1038/s42003-022-03516-4

**Published:** 2022-06-09

**Authors:** Hiroyuki Ueno, Takuya Hoshino, Wakako Yano, Sayaka Tsukioka, Takamasa Suzuki, Shoki Hara, Yoshio Ogino, Khoon Tee Chong, Tatsuya Suzuki, Shingo Tsuji, Hikaru Itadani, Ikuo Yamamiya, Yoshihiro Otsu, Satoshi Ito, Toshiya Yonekura, Miki Terasaka, Nozomu Tanaka, Seiji Miyahara

**Affiliations:** Discovery and Preclinical Research Division, Taiho Pharmaceutical Co., Ltd., Tsukuba, Ibaraki, Japan

**Keywords:** Pharmacology, Cancer metabolism, Targeted therapies, Pharmacodynamics

## Abstract

Ribonucleotide reductase (RNR) is composed of two non-identical subunits, R1 and R2, and plays a crucial role in balancing the cellular dNTP pool, establishing it as an attractive cancer target. Herein, we report the discovery of a highly potent and selective small-molecule inhibitor, TAS1553, targeting protein-protein interaction between R1 and R2. TAS1553 is also expected to demonstrate superior selectivity because it does not directly target free radical or a substrate binding site. TAS1553 has shown antiproliferative activity in human cancer cell lines, dramatically reducing the intracellular dATP pool and causing DNA replication stress. Furthermore, we identified SLFN11 as a biomarker that predicts the cytotoxic effect of TAS1553. Oral administration of TAS1553 demonstrated robust antitumor efficacy against both hematological and solid cancer xenograft tumors and also provided a significant survival benefit in an acute myelogenous leukemia model. Our findings strongly support the evaluation of TAS1553 in clinical trials.

## Abstract (web summary)

A small-molecule protein-protein interaction inhibitor of ribonucleotide reductase subunit, TAS1553, is shown to inhibit growth of both hematological and solid cancer xenograft tumors following oral administration in mice.

## Introduction

The Warburg effect of aerobic glycolysis is a known hallmark of cancer^1^. Most of the cancer cells consume high amounts of glucose to supply not only energy sources such as ATP, but also many metabolites required for tumor proliferation, such as lipids, amino acids, and nucleic acids. Among various intracellular metabolic processes, the nucleic acid metabolism has long been targeted by several antimetabolites, such as nucleoside analogs, for cancer treatment^2^. Therefore, targeting nucleic acid metabolism is considered a well-validated strategy for cancer treatment.

Nucleic acid metabolism involves the production of important metabolites such as ribonucleotide triphosphate (NTP), which is an essential metabolite for RNA synthesis and an energy source, and deoxyribonucleotide triphosphate (dNTP), which is a building block for DNA^3^. Over two decades ago, it was shown that dNTP pools were larger in tumor cells than in normal cells^4^, indicating the importance of synthesizing dNTP for tumor cells. This idea has been supported by findings that almost all nucleoside analogs exerted antitumor effects via utilizing and/or targeting the metabolic pathways required for dNTP production^2,3^. In addition, recent reports have shown that genes involved in dNTP synthesis were significantly upregulated in tumor tissue compared to those in the normal tissue in clinical patient samples^5,6^. These facts imply that inhibiting dNTP production is a promising strategy for cancer treatment.

Ribonucleotide reductase (RNR) is a rate-limiting enzyme for *de novo* dNTP production, comprising α and β subunits called R1 and R2, respectively. The active mammalian form of the RNR holo complex consists of α_6_β_n_ (*n* equals 1, 2, 3 dimers of R2 subunit)^7–10^. It catalyzes the reaction in which nucleotide diphosphate (NDP) is reduced to deoxyribonucleotide diphosphate (dNDP), which is further converted to dNTP by NDP Kinase^11^. The RNR activity is strictly regulated, mainly by the expression level of its R2 subunit^12^, for a controlled cell proliferation^3^. Importantly, overexpression of R2 subunits causes tumor initiation and progression in mice^13^, and the knockdown of either R1 or R2 subunit inhibits tumor cell growth in preclinical models^14,15^. Furthermore, R2 is highly upregulated in tumor tissues compared with that in normal tissues in clinical samples^6^ and correlates with poor survival in many cancer types^16–18^.

Given the critical function of RNR in cancer cells, it is clear that RNR has long been recognized as a promising target for treating cancer^11,19^ and several compounds, such as radical scavengers, iron chelators, and nucleoside analogs, have been developed as RNR inhibitors in the anti-cancer compounds field. Hydroxyurea (HU) was the earliest-recognized RNR inhibitor and has a 30-year history as a cancer therapeutic agent^19^. HU is a radical scavenger with low inhibitory activity against RNR and has various off-target effects through its radical quenching capability^19^ and nitric oxide production^20^. Triapine (3-AP), which is an iron chelator in clinical trials and has stronger RNR inhibitory activity than hydroxyurea^19,21^, also affects hemoglobin homeostasis leading to serious side effects including hypoxia and methemoglobinemia^22^. A variety of nucleoside analogs also inhibit RNR, and some of them have been used in cancer chemotherapies^11,19^. The best known amongst them is gemcitabine (2′,2′-difluorodeoxycytidine), which, once metabolized to its diphosphate form, functions as a pseudosubstrate of NDP, occupies the catalytic site on R1 via irreversibe binding and hence inhibits RNR^23,24^. Other nucleoside analogs, such as fludarabine and clofarabine, are metabolized to their triphosphate form and then inhibit RNR by targeting an allosteric site on R1^11^. However, the mechanisms of action of the nucleoside analogs include not only RNR inhibition but also other effects such as DNA chain termination, indicating that these analogs are not specific RNR inhibitors^24^. Therefore, the importance of RNR inhibition for anti-tumor efficacy has not fully been elucidated althought HU and the nucleoside analogues have been approved and used as cornerstone therapies.

The search for more potent RNR inhibitors led to a discovery of aromatically substituted thiazole derivatives such as COH29, which binds to R2^25^. COH29 has a 3, 4-dihydroxybenzene group; therefore, it seems to act as a radical quencher, such as HU, suggesting that its effects will extend beyond RNR inhibition. Additionally, some novel small moleules targeting RNR have been reported, but there is no evidence that these molecules are available for in vivo experiments^26–29^.

The development of a new class of more specific inhibitors has led to a production of peptide derivatives that corresponded to the C-terminal residues of R2 and interfered with the oligomeric structure of RNR via binding to R1 and then blocking protein-protein interaction^30–33^. In 1986, two groups reported a nonapeptide corresponding to the C-terminus of the HSV-1 RNR small subunit (equivalent to R2 subunit), with an ability to prevent the formation of active holoenzyme by competing with the small subunit for binding to the large subunit (equivalent to R1 subunit), thus inhibiting HSV RNR activity^30,31^. Through the same mechanism, mammalian RNR is also inhibited by P7 heptapeptide (N-AcFTLDADF), corresponding to the C-terminus of the R2 subunit^32,33^, or P6 peptidomimetic^34^. These findings imply that disrupting the interactions between R1 and R2 is an emerging alternative to obtain more selective and potent inhibitors since an RNR subunit interaction inhibitor does not directly target free-radical or substrate binding sites, which might lead to undesired off-target effects as described above. However, peptides are intrinsically considered to lack favorable drug-like properties, including membrane permeability, large molecular size, immunogenic potential, and short half-life in the plasma due to susceptibility to enzymatic degradation. Thus, to our knowledge, no small molecule or peptide was reported to specifically bind to R1 or R2 with the potency to disrupt R1-R2 interaction at the cellular level. In this study, we describe the discovery of TAS1553, a novel and potent small-molecule RNR subunit protein-protein interaction inhibitor, and its profile in the preclinical evaluation.

## Results

### Design concept of protein–protein interaction inhibitor against human RNR

**Fig. 1:**
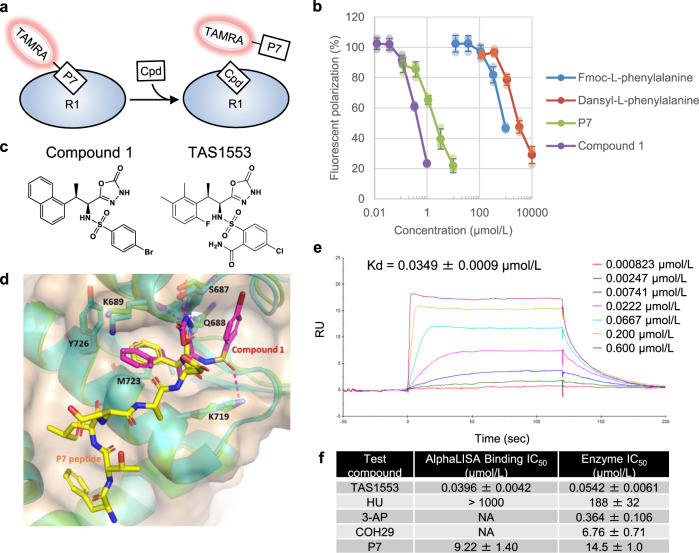
Design and identification of the small molecule compound TAS1553 blocking the interaction between RNR subunits. **a** A schematic representation of the fluorescent polarization binding assay. TAMRA-labeled P7 heptapeptide was used as a probe. Cpd compound. **b** Hit compounds show the displacement of labeled P7 from human R1, measured by the fluorescent polarization binding assay. Data are presented as mean ± SD obtained from three independent experiments. **c** Chemical structures of compound 1 and TAS1553. **d** Superposition of the X-ray co-crystal structure of human RRM1 (green) with compound 1 (magenta) (PDB ID: 6L3R) to the previously determined X-ray co-crystal structure of *Saccharomyces cerevisiae* RNR1 (cyan) with P7 peptide (yellow) (PDB ID: 2ZLF). The residue numbers and protein surface shown here are those of hRRM1. **e** SPR analysis for the binding of TAS1553 to human R1 captured onto Series S Sensor Chip NTA. Representative sensorgram of the TAS1553 at 0.000823–0.600 µmol/L is presented. Kd value is obtained from three independent experiments. **f** IC_50_ values of RNR inhibitors for the AlphaLISA binding and activity of RNR. IC_50_ (mean ± SD) is determined from three independent experiments performed in triplicate.

Aiming to identify a novel small molecule disrupting RNR subunit interaction, we created a small focused library that included various amino acid derivatives, based on the report that Fmoc-L-phenylalanine competes with P7 heptapeptide for binding to R1, with a weak Kd value, determined using the P7-coupled sepharose affinity column^35^. We then established a fluorescent polarization (FP) binding assay using R1 and fluorescence tagged P7 to enable high-throughput screening (Fig. 1a) in which the unlabeled P7 showed a concentration-dependent inhibition with IC_50_ of 2.49 ± 0.44 µmol/L (Fig. 1b). By screening the library, we found that Fmoc-l-phenylalanine and other amino acids such as Dansyl-l-phenyl alanine had a weak but concentration-dependent inhibition activity with IC_50_ of 934 ± 35 µmol/L and 3340 ± 540 µmol/L, respectively. The small size and simple structure of these amino acids make them an attractive starting point for hit expansion. Subsequent medicinal chemistry efforts focused on improving their binding, enzymatic and cellular activity that led to the formation of the unique β-methyl-α-amino acid derivative (compound 1), which showed significantly improved binding inhibition activity (IC_50_ of 0.527 ± 0.021 µmol/L) with a potency ~2000-fold higher than that of Fmoc-l-phenylalanine (Fig. 1b, c). Subsequently, we analyzed the X-ray cocrystal structure of compound 1 with R1 to examine the binding site and the binding mode of the compound (PDB ID: 6L3R) at a resolution of 2.0 Å, which provided the structural information on a non-peptide, small molecule inhibitor bound to R1, enabling structure-based lead optimization. The result clearly demonstrated the defined binding mode in which compound 1 was accommodated in the C terminus R2 binding site at R1, and it was also consistent with previously determined binding site for C terminus of P7^34^ (Fig. 1d). Interestingly, a part of the molecule (4-bromophenyl group) is found to accesse to small hydrophobic poket, leading to a key differenrence as compared with the corresponding region of P7. The oxadiazolone ring forms hydrogen bonds with Ser687, Gln688, and Lys689, whereas the left naphthyl group sits in a small hydrophobic cleft formed by Lys689, Met723, and Tyr726. The carbonyl group of the sulfonamide forms hydrogen bonds with Lys719. Guided by the crystal structures of R1 bound with compound 1, several highly potent nanomolar inhibitors were identified through lead optimization (details to be published elsewhere). Among them, we selected TAS1553 as a preclinical candidate since the compound had a well-balanced compound profile (Fig. 1c).

### TAS1553 is a potent protein-protein interaction inhibitor against human RNR

The binding affinity of TAS1553 to the R1 subunit was investigated using surface plasmon resonance (SPR) binding analysis. The Kd value of TAS1553 for R1 is 0.0349 ± 0.0009 µmol/L (Fig. 1e). To elucidate whether TAS1553 has the ability to block the interaction of R1 and R2, we established an AlphaLISA assay using recombinant R1 and R2, where P7 inhibited the interaction with an IC_50_ value of 9.22 ± 1.40 µmol/L. The assay revealed that TAS1553 inhibited the interaction between R1 and R2 in a concentration-dependent manner with an IC_50_ value of 0.0396 ± 0.0042 µmol/L (Fig. 1f and Supplementary Fig. 1). In contrast, HU did not show any inhibitory effect against the interaction under 1000 µmol/L. The IC_50_ value for 3-AP and COH29 could not be determined due to their direct inhibitory effect on the AlphaLISA system independently on RNR, while TAS1553, P7, and HU did not show the direct effect on the system (Fig. 1f and Supplementary Fig. 1). Furthermore, immunoprecipitation assay using recombinant enzyme also showed inhibitory effect of TAS1553 on interaction between R1 and R2 (Supplementary Fig. 2). Next, we evaluated the inhibitory effect of TAS1553 against the enzymatic activity of RNR, consisting of R1 and R2 recombinant proteins. RNR activity was determined by its ability to reduce cytidine diphosphate (CDP) to deoxycytidine diphosphate (dCDP), based on a previous report^36^. The results showed that TAS1553 inhibited the enzymatic activity of RNR in a concentration-dependent manner (Supplementary Fig. 3). Notably, TAS1553 demonstrated the lowest IC_50_ value of 0.0542 ± 0.0061 µmol/L among those of the evaluated compounds (Fig. 1f). In addition to the inhibitory effect on RNR, TAS1553 was evaluated in a Eurofins LeadProfilingScreen panel assay including 68 proteins, such as ion channels, transporters, nuclear receptors, and G protein-coupled receptors, to examine its potential adverse activity. Importantly, no significant inhibition by TAS1553 was observed, even at a concentration of 10 μmol/L (defined as >50% inhibition for each target protein) (Supplementary Table 1). These analyses, therefore, demonstrate that TAS1553 shows highly potent RNR inhibitory activity and has clean selectivity profile againt at least 68 proteins.

### TAS1553 shows a wide-range of antiproliferative activity

The growth inhibitory effect of TAS1553 was determined by calculating the GI_50_ value against human solid and hematological cancer cell lines. As seen in Supplementary Table 2, TAS1553 showed antiproliferative activity against both solid and hematological human cancer cell lines. The GI_50_ values for TAS1553 ranged from 0.228 to 4.15 μmol/L and were lower than those for HU in all cell lines.

### TAS1553 inhibits the protein–protein interaction between R1 and R2 in cultured cells

**Fig. 2:**
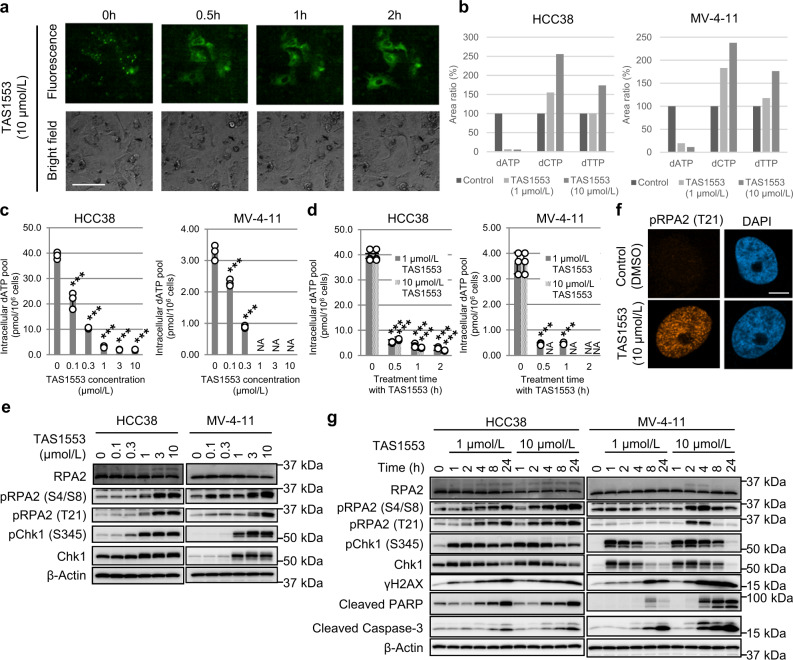
Effect of TAS1553 on RNR subunit interaction, dNTP pool, DNA replication stress, and apoptosis. **a** Inhibition of intracellular protein-protein interaction between R1 and R2 by TAS1553. HCC38 cells co-transfected with cDNAs encoding AG-tagged R1 and Ash-tagged R2 were treated with TAS1553 for 2 h, followed by incubation with drug-free medium for 1 h. Fluorescence and bright-field images were obtained at the indicated timepoints. R1-R2 interaction is visible as green dots in fluorescence images. Scale bar, 100 µm. **b** Effect of TAS1553 on intracellular dNTP pool in HCC38 and MV-4-11 cells. Relative changes of dATP, dCTP, and dTTP peak area in HCC38 and MV-4-11 cells treated with TAS1553 for 2 h or DMSO as control. Intracellular metabolites were measured by LC-MS/MS. **c** Concentration-dependent changes of the dATP pool in HCC38 cells and MV-4-11 cells treated with TAS1553 for 2 h. **d** Time-dependent changes of the dATP pool in HCC38 cells and MV-4-11 cells treated with TAS1553. Data are presented as the mean ± SD (*N* = 3). NA not applicable. ***P < 0.001 versus control (0 µmol/L or 0 h). **e** Induction of DNA replication stress and apoptosis by TAS1553 in HCC38 and MV-4-11 cells. Concentration-dependent effect of TAS1553 on DNA replication stress in HCC38 cells and MV-4-11 cells. The cells were incubated with TAS1553 and harvested 2 h post-treatment. The expression of indicated proteins was analyzed by immunoblotting. **f** Immunofluorescence analyses in HCC38 cells treated with TAS1553 at 10 µmol/L for 2 h. Representative confocal microscopy images; pRPA2 (Thr21) (red) and DAPI (blue). Scale bar, 10 µm. **g** Time-dependent effect of TAS1553 on DNA replication stress and apoptosis in HCC38 cells and MV-4-11 cells.

We examined whether TAS1553 inhibited the interaction of RNR subunits in cultured cells. Fluorescent‐based technology detecting protein-protein interaction (Fluoppi) analysis utilizing Azami-green tag (AG-tag) and Assembly-helper tag (Ash-tag)^37^ was used to monitor the intracellular interaction between R1 and R2. Plasmids encoding N-terminally AG-tagged hR1 (75–742 aa) and N-terminally Ash-tagged hR2 were co-transfected into the breast cancer cell line HCC38. Fluorescence imaging revealed foci, representing intracellular interactions between R1 and R2, in the pre-treatment samples, 24 h post-transfection (Fig. 2a). Although DMSO or HU treatment did not affect the foci, the treatment with TAS1553 reduced their numbers and increased the dispersed staining within 30 minutes in HCC38 cells and H460 cells (Fig. 2a and Supplementary Fig. 4). Because the recovery of foci disappearance was observed 1 h post washout of TAS1553, it appeared that TAS1553 rapidly and reversibly abrogated the protein-protein interaction between R1 and R2 in treated cells.

### TAS1553 reduces intracellular dATP pool

Next, we examined the RNR inhibitory effect of TAS1553 in cells by measuring dNDP pools which are direct products of RNR. However, all dNDP pools could not be detected using LC-MS/MS due to detection limit. Then we determined whether TAS1553 affected the intracellular dNTP pools as surrogate marker for RNR inhibition in cells. LC-MS/MS could not measure dGTP pool due to masking dGTP peak by ATP, as previously reported^38^; thus, dATP, dCTP and dTTP pools were analyzed. HCC38 and MV-4-11, an acute myelogenous leukemia (AML) cell line, were exposed to TAS1553, and the peak area of each dNTP was determined using cell extracts. TAS1553 dramatically reduced the dATP area in both cell lines (Fig. 2b), while it increased the dCTP and dTTP areas probably due to activation of nucleoside salvage pathway^39^. These results are consistent with the previous reports showing that the dATP pool was the most sensitive dNTP pool affected by RNR inhibition^21,40^.

Next, we evaluated both concentration-dependent and time-dependent effects of TAS1553 on the dATP pool in HCC38 and MV-4-11 cell lines using absolute quantification. After 2 h of TAS1553 exposure, there was a concentration-dependent reduction of intracellular dATP pool in both cell lines (Fig. 2c). Furthermore, the intracellular dATP pool was reduced within 30 minutes when treated with TAS1553 at 1 or 10 µmol/L in both cell lines (Fig. 2d), consistent with its inhibitory effect on the protein-protein interaction (Fig. 2a). Because the GI_50_ values for TAS1553 against HCC38 and MV-4-11 cells were 0.352 µmol/L and 0.393 µmol/L, respectively, it seems likely that TAS1553 shows growth inhibitory effects associated with dATP pool reduction via abrogating the subunit interaction of RNR. Notably, at least until 2 h, TAS1553 treatment did not affect the intracellular ATP pool remarkably, even at 10 µmol/L (Supplementary Fig. 5), suggesting that TAS1553 inhibited RNR without any impact on the ribonucleotide synthesis pathway or energy production.

### TAS1553 induces the replication stress and apoptosis

Because dATP is a critical metabolite for DNA replication, we next examined whether TAS1553 caused DNA replication stress^41^. We evaluated phosphorylation of Chk1 at Ser345 in HCC38 and MV-4-11 cells by treating them with TAS1553 for 2 h. We found that the phosphorylation of Chk1 was markedly induced when treated with TAS1553 at 1 µmol/L in both cell lines (Fig. 2e). Furthermore, we found that TAS1553 treatment induced phosphorylation of RPA2 at Ser4, Ser8, and Thr21 at 3 µmol/L in both cell lines. Because phosphorylated RPA2 at Thr21 binds to single-strand DNA at the stalled replication fork^42^, TAS1553 may cause the replication fork stalling. To test this possibility, we analyzed the nuclear localization of phosphorylated RPA2 at Thr21. Immunofluorescence staining revealed that TAS1553 treatment at 10 µmol/L caused foci formation of phosphorylated RPA2 at Thr21 in the nuclei (Fig. 2f), suggesting the recruitment of RPA2 at the stalled replication fork. Thus, these results indicated that TAS1553 caused DNA replication stress as well as fork stalling via dATP pool depletion.

Next, we examined the effect of TAS1553 on apoptosis induction in HCC38 and MV-4-11 cells. We treated TAS1553 at 1 or 10 µmol/L for 24 h and analyzed the time-dependent change of cleaved PARP and cleaved caspase-3 (Fig. 2g). Compared with control cells (0 h), phosphorylation of Chk1 at Ser345 was increased after 1 h of TAS1553 treatment at 1 and 10 μmol/L in both cell lines. Phosphorylation of RPA2 was also detected after 2 h of treatment with TAS1553 at 10 μmol/L. Following the induction of replication stress and fork stalling, cleaved PARP and cleaved caspase-3 levels were increased in both cell lines after 4 h of TAS1553 treatment at 10 μmol/L or after 8 h of treatment at 1 μmol/L.

Furthermore, we asked whether cellular events caused by TAS1553, such as induction of replication stress, apoptosis, or proliferation inhibition, are dependent on RNR inhibition. Then we firstly examined the effects of TAS1553 in cells treated with siRNA against R1. Because full knockdown of R1 affected cell proliferation, we treated the siRNA at low concentration to achieve partial knockdown of R1. Then, TAS1553 showed growth inhibitory effect and caspase induction at lower concentration in R1-knockdowned cells than in control cells (Supplementary Fig. 6). Additionally, it was observed that supplementation of four deoxynucleosides canceled the effect of TAS1553 on replication stress and apoptosis induction (Supplementary Fig. 7). Thus, these findings strongly suggested that TAS1553 inhibited RNR, then caused dATP pool depletion followed by replication stress, and finally triggered apoptosis.

### SLFN11 promotes apoptosis caused by TAS1553

**Fig. 3:**
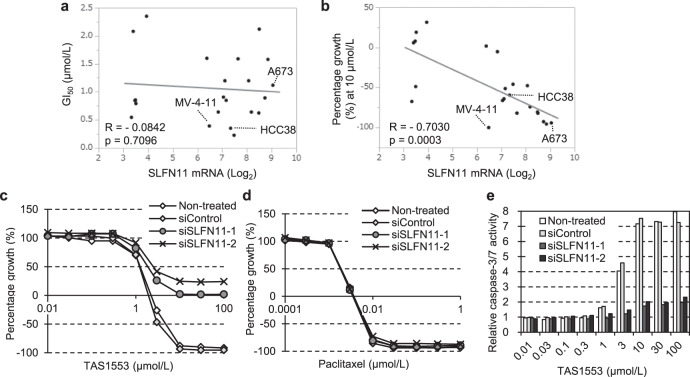
Involvement of SLFN11 in apoptosis induction by TAS1553. **a** Scatterplot indicating the correlation between SLFN11 expression and TAS1553 GI_50_ in 22 cell lines. **b** Scatterplot idicating the correlation between SLFN11 expression and percentage cell growth (%) at 10 µmol/L of TAS1553 in the same cell lines as mentioned in **a**. **c**, **d** Effects of TAS1553 (**c**) and paclitaxel (**d**) on the proliferation of A673 cells treated with siRNA against SLFN11. The cells were treated with the test compound for 72 h 2 days after treatment with each siRNA. **e** Caspase3/7 activation by TAS1553 in A673 cells treated with siRNA against SLFN11.

The identification of a predictive biomarker is essential to maximize the clinical benefit of TAS1553. In some reports using biological tools, SLFN11 has been identified and validated as a factor to sensitize cancer cells to DNA damaging agents and replication inhibitors^43^, as well as to RNR inhibition^44^. These findings prompted us to investigate whether SLFN11 expression correlated with the sensitivity to TAS1553 in cell lines listed in Supplementary Table 2 (except for BHK-89, Ca9-22, and DOK, due to the absence of public gene expression data). However, we could not find any correlation between SLFN11 mRNA and GI_50_ values for TAS1553 (Fig. 3a). We then hypothesized that SLFN11 expression correlated with the cytotoxic effects of TAS1553 and analyzed the association between SLFN11 mRNA and the relative growth percent of cells at 10 µmol/L, where apoptosis induction was observed in the two cell lines (Fig. 2g). In this analysis, we detected a significant correlation between SLFN11 mRNA and relative growth percentage at 10 µmol/L (Fig. 3b). To determine whether SLFN11 directly affected the cytotoxic property of TAS1553, we examined the effect of SLFN11 knockdown using siRNA on the cytotoxicity of TAS1553 in an Ewing sarcoma cell line A673 (which had the highest SLFN11 expression among the 22 cell lines). SLFN11 knockdown significantly suppressed the cytotoxic effect of TAS1553 but not the growth inhibitory effect, although the knockdown did not affect the cytotoxicity of paclitaxel (Fig. 3c, d and Supplementary Fig. 8). Furthermore, we observed that the suppression of TAS1553-induced caspase-3/7 activation in A673 cells transfected with siRNA against SLFN11 (Fig. 3e). These results were consistent with the apoptosis induction by TAS1553 in both MV-4-11 and HCC38, which had high SLFN11 expression (Fig. 3b). Thus, SLFN11 appears to sensitize tumor cells to TAS1553 via promoting apoptosis.

### Characterization of TAS1553 using in vivo models

**Fig. 4:**
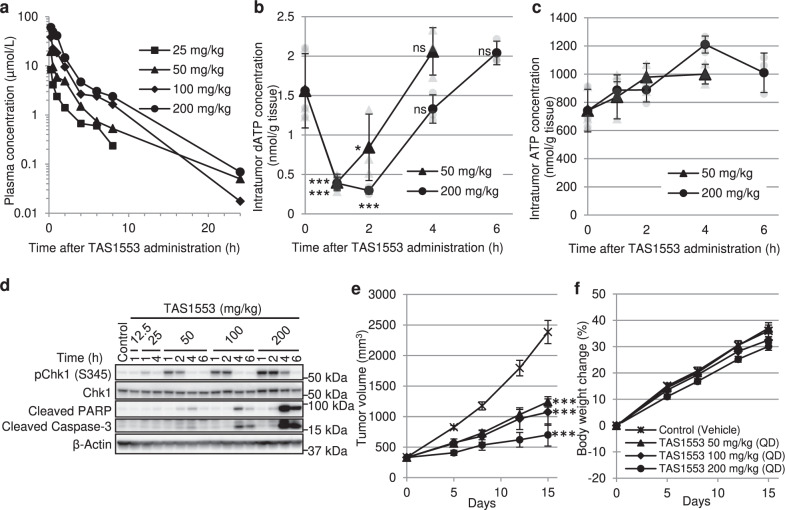
In vivo pharmacodynamic effect and antitumor efficacy of TAS1553. **a** Plasma concentration of TAS1553 in nude rats after oral administration. Average concentrations shown at each point (*N* = 2). **b**, **c** Effect of TAS1553 oral administration on the intratumoral dATP pool (**b**) and ATP pool (**c**) in MV-4-11 xenograft tumors. Data are presented as the mean ± SD (*N* = 3). **d** MV-4-11 xenograft tumors were collected and the expression of indicated proteins was analyzed by immunoblotting. **e**, **f** Inhibition of tumor growth (**e**) and body weight change (**f**) in rats bearing MV-4-11 xenografts over a 14-day treatment with oral TAS1553. Data are presented as the mean ± SEM (*N* = 5). *P < 0.05 and ***P < 0.001 versus control group. ns not statistically significant.

The above in vitro profile prompted us to evaluate the antitumor activity of TAS1553 in an in vivo model. First, we analyzed the exposure of TAS1553 when administered orally. A single dose of TAS1553 led to its dose-dependent rise in plasma in nude rats (Fig. 4a and Supplementary Table 3).

To determine whether TAS1553 achieved RNR inhibition in an in vivo model, we examined the effect of a single dose of orally administered TAS1553 on intratumoral dATP pool, DNA replication stress, and apoptosis in rats that were subcutaneously implanted with a xenograft of MV-4-11 cell line. TAS1553 administration showed a significant reduction in the intratumoral dATP pool, but not the ATP pool, 1 and 2 h post-administration at both 50 mg/kg and 200 mg/kg (Fig. 4b, c). The decline in the intratumoral dATP pool recovered 4 h after the administration of both doses. We also evaluated the DNA replication stress and apoptosis induction in tumors following TAS1553 administration. Consistent with the modulation of the dATP pool, the single administration of TAS1553 (50 mg/kg or more) caused the phosphorylation of Chk1 at Ser345 1 and 2 h post-administration. Furthermore, TAS1553 administration also led to induction in cleaved PARP and cleaved caspase-3 levels, following to Chk1 phosphorylation (Fig. 4d).

Next, to examine the antitumor activity of TAS1553, we administered TAS1553 once daily for 14 days to rats bearing the MV-4-11 xenograft. At TAS1553 doses of 50, 100, and 200 mg/kg, the ratios of the mean tumor volume in the treated group/control group (T/C) on Day 15 were 52.0, 45.0, and 29.4%, respectively (Fig. 4e), all differences being statistically significant. The analysis of the mean body weight change revealed that none of the groups showed any dramatic weight loss during the study period compared to the control group (Fig. 4f). These findings indicated that TAS1553 demonstrated antitumor activity associated with RNR inhibition in a dose-dependent manner.

### TAS1553 demonstrates antitumor activity in both hematologic and solid tumor models

**Fig. 5:**
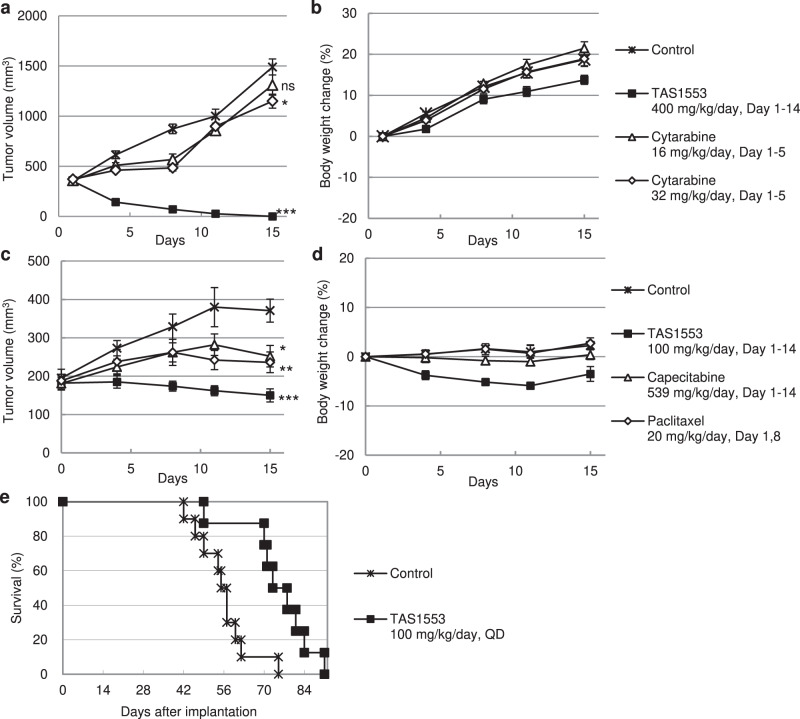
Anti-tumor effect of TAS1553 in multiple models. **a**, **b** Inhibition of tumor growth (**a**) and body weight change (**b**) in rats bearing MV-4-11 xenografts. TAS1553 was orally administered at 400 mg/kg for 14 days, and cytarabine was administered intravenously at 16 or 32 mg/kg for 5 consecutive days (Day 1–5). Data are presented as the mean ± SEM (*N* = 5). ns not significant. **c**, **d** Inhibition of tumor growth (**c**) and body weight change (**d**) in mice bearing HCC38 xenografts. TAS1553 and capecitabine were orally administered for 14 days once daily at 100 mg/kg and 539 mg/kg, respectively. Paclitaxel was administered intravenously once a week at 20 mg/kg. Data are presented as the mean ± SEM (*N* = 6). **e** Survival benefit of daily oral administration of TAS1553 in the systemic model. Kaplan–Meier survival curve of mice inoculated with mice bone marrow cells harboring human *MLL-AF9* fusion gene and *Luciferase* via the tail vein. Eleven days after implantation, mice were administered with vehicle (*N* = 10) or TAS1553 (*N* = 8) at 100 mg/kg once daily. *P < 0.05, **P < 0.01, and ***P < 0.001 versus control group. ns not statistically significant.

Next, we evaluated the antitumor activity of a high-dose administration of TAS1553 and cytarabine in rats bearing MV-4-11 xenografts. When administered from the tail vein, the AUC_0-8h_ of cytarabine at 16 mg/kg was 75.76 µmol*h, which is almost comparable to the exposure level of high-dose cytarabine therapy at 2 g/m^2^ during AML treatment^45^. The rats were treated with cytarabine for 5 days at 16 and 32 mg/kg. Once-daily treatment with TAS1553 at 400 mg/kg resulted in tumor regression and finally achieved tumor disappearance in all the treated animals (Fig. 5a) without a reduction in body weight (Fig. 5b). Cytarabine at 32 mg/kg showed minimal but statistically significant antitumor activity with a T/C ratio of 77.4%. In addition to the hematologic tumor, we evaluated the antitumor activity of TAS1553 in mice implanted with HCC38 cell line as a solid tumor model. TAS1553, administered at a dose of 100 mg/kg daily, showed a statistically significant antitumor activity with a T/C ratio of 40.4%, while capecitabine at 539 mg/kg daily (maximum tolerated dose)^46^ and paclitaxel at 20 mg/kg weekly^47^ showed statistically significant antitumor activity with T/C ratios of 67.9 and 63.6%, respectively (Fig. 5c, d). Finally, TAS1553 was evaluated for its survival benefit in a mouse model. To achieve this, we established mice bone marrow cells harboring the human *MLL-AF9* fusion gene, which is known as a driver gene for AML in humans as well as mice^48^. Mice were transplanted with the cells through the tail vein and the treatment with TAS1553 was started 11 days post-implantation. When administered daily at 100 mg/kg, TAS1553 significantly prolonged median survival time (75.5 days) compared with the control group (56 days), with an increase in the life span of 34.8% (Fig. 5e). Thus, TAS1553 achieved antitumor activity against both hematologic and solid tumor in in vivo tumor models.

## Discussion

RNR has been recognized as a promising therapeutic target for cancer treatment^11,19^. However, current RNR inhibitors used in the clinic have pharmacological limitations, mainly due to poor affinity against RNR and/or poor selectivity, which significantly restricts their use as oncology therapeutics. Starting from weak but small amino acid derivatives, we identified initial lead compound 1, which had ~2000-fold increased activity over hit amino acid derivatives. We examined the crystal structure of R1 with compound 1 to elucidate the binding site as well as the binding mode of the compound. Further structure-activity relationship studies of compound 1 using structural information led to the identification of TAS1553 which was highly potent RNR inhibitor with a clean selectivity profile as validated by Eurofins LeadProfilingScreen panel assay.

Although this study demonstrated that TAS1553 inhibits RNR activity via abrogating protein-protein interaction between R1 and R2, it still remains unclear how TAS1553 actually interacts with and modulates the active form of RNR holo complex composed of α_6_β_n_^7–10^. In addition, compared with previous reports, the activity of RNR used in this study seems to be limited probably due to including a certain amount of apo form of R2 subunit^10,49^. Detailed inhibitory mechanism of TAS1553 is needed to be clarified in future studies with fully active enzyme.

Consistent with the physiological function of RNR, TAS1553 showed wide-ranging antiproliferative activity against several types of cancer cell lines. Importantly, a report suggested that AML cell lines THP-1 and MV-4-11 were resistant to nucleoside analogs, including cytarabine, due to the high expression of SAMHD1, which is a hydrolase that degrades active and triphosphorylated metabolites of the analogs^50^. However, TAS1553 showed antiproliferative activity against the two cell lines at comparable levels against other cell lines (Supplementary Table 2). Furthermore, TAS1553 caused tumor regression in rats bearing MV-4-11 xenografts, which showed poor response to cytarabine treatment (Fig. 5a). Given the critical role of RNR in cancer and the direct inhibition of RNR by TAS1553, these findings suggest that TAS1553 shows antitumor activity in tumors that are refractory or resistant to conventional anticancer therapies.

Furthermore, we identified SLFN11 as a factor to promote apoptosis induction by TAS1553. SLFN11 has previously been established as a factor to augment antitumor activity of DNA replication inhibitors, such as Topoisomerase I inhibitor and PARP inhibitor, at least in preclinical models^43,51^. In response to DNA replication stress, SLFN11 binds to replication forks via interaction with RPA and blocks replication by unwinding the chromatin^52^. As shown in Fig. 2, TAS1553 caused foci formation of phosphorylated RPA2 in the nuclei. Therefore, SLFN11 seems to be recruited in stressed replication forks formed by TAS1553 and mediate chromatin opening and apoptotis similary to the topoisomerase I inhibitor, although its specific mechanism should be addressed in more detail. In addition to findings in the preclinical setting, it has been suggested that SFLN11 predicted a good response to platinum-based therapy or to PARP inhibitors in the clinical setting^51,53^. Based on our data, it seems that SLFN11 expression is also a predictive biomarker of sensitivity towards TAS1553 in the clinical setting, and SLFN11 augments the clinical benefit of TAS1553. This hypothesis will be examined in the clinical trial for TAS1553.

TAS1553 treatment increased the intracellular dCTP pool and dTTP pool, while it reduced the dATP pool (Fig. 2b). This result is consistent with reports showing that the inhibition of *de novo* dNTP pathway through RNR inhibition mediates the activation of the salvage pathway, especially for dCTP production^39^. Given that anticancer nucleoside analogs are activated through the salvage pathway^2^, it is suggested that TAS1553 shows antitumor efficacy not only in monotherapy but also in combination with nucleoside analogs, especially dCyd analogs. The idea is supported by a report showing the enhanced cytotoxicity of gemcitabine and cytarabine in combination with 3-AP in cell lines^54^. Furthermore, the utilization of dATP for DNA repair suggests that TAS1553 also augments the antitumor efficacy of DNA damaging agents. p53R2, another small subunit of RNR, is reported to be induced by p53 in response to DNA damage, to supply dNTPs for DNA repair^55^. Importantly, C-termininus of p53R2 is identical to that of R2, and P7 peptide inhibits RNR activity of both R1/R2 and R1/p53R2^56^. These findings imply that TAS1553 inhibits dATP synthesis not only in DNA replication, but also in DNA repair through dual inhibition of R1/R2 and R1/p53R2. Therefore, future studies are required to clarify the potential of TAS1553 for combination therapy with other chemotherapies. Thus, the clinical testing of TAS1553 will provide the ultimate proof of the usefulness of this therapeutic strategy for the treatment of cancers.

## Methods

### Chemical compounds

Compound 1 {4-bromo-*N*-[(1*S*,2*R*)-2-(naphthalen-1-yl)-1-(5-oxo-4,5-dihydro-1,3,4-oxadiazol-2-yl)propyl]benzenesulfonamide} and TAS1553{5-chloro-2-{*N-*[(1*S*,2*R*)-2-(6-fluoro-2,3-dimethylphenyl)-1-(5-oxo-4,5-dihydro-1,3,4-oxadiazol-2-yl)propyl]sulfamoyl}benzamide} were synthesized at Taiho Pharmaceutical Co., Ltd. as described in Supplementary Note 1. Fmoc-l-Phenylalanine and Dansyl-l-Phenylalanine were obtained from Tokyo Chemical Industry Co., Ltd. P7 heptapeptide (N-AcFTLDADF), HU, 3-AP, and COH29 were obtained from Peptide Institute, Inc., Abcam plc., Cayman CHEMICAL, and Mitsui Chemicals, Inc., respectively. dATP and ATP were obtained from Thermo Fisher Scientific, Inc. IS-dATP (#NLM-6215-SL-10) and IS-ATP (#DLM-7514-CA-20) were obtained from Cambridge Isotope Laboratories, Inc. and used for LC-MS/MS analysis. Cytarabine hydrochloride crystalline, capecitabine, and paclitaxel were obtained from Sigma-Aldrich Japan, F. Hoffmann-La Roche Ltd, and FUJIFILM Wako Pure Chemical Corporation, respectively.

### Cell lines

The Ca9-22, HEL, NUGC-3, and RPMI8226 cell lines were obtained from the Japanese Collection of Research Bioresources. The 786-O, CFPAC-1, DU145, HCC1599, HCC1806, HCC38, HCT116, HL-60, K-562, MSTO-211H, MV-4-11, NCI-H460, NCI-H2170, and THP-1 cell lines were obtained from the ATCC. The A2780, COLO 792, and DOK cell lines were obtained from the European Collection of Authenticated Cell Cultures. The A549, A673, BHL-89, and MCF-7 cell lines were obtained from DS Pharma Biomedical Co., Ltd. Short-tandem repeat-based DNA profiling was used to reauthenticate cell lines. All cell lines were tested for mycoplasma contamination.

### Genetically engineered cell lines

Bases 24–4208 encoding the residues 1–1395 of human KMT2A (Accession NM_001197104) and bases 1722–1994 encoding the residues 479–568 of human MLLT3 (Accession NM_004529) were amplified using PCR from human cDNA library and cloned into pMYs-IRES-Puro Retroviral Vector (pMYs-MLL-AF9-IRES-Puro). pMYs-MLL-AF9-IRES-Puro and pMYs-IRES-Neo Retroviral Vector, including *Luciferase* (pMYs-Luciferase-IRES-Neo), were packaged into viruses using the GP2-293 cell line (Retro-X™ Universal Packaging System, Clontech Laboratories, Inc.). c-Kit positive bone marrow cells were obtained from B6N (IGS): C57BL/6NCrl mice and infected with the packaged viruses, followed by clone selection using puromycin and neomycin. The cells were cultured with IMDM medium including 20% FBS, 10 ng/mL GM-CSF, 10 ng/mL IL-3, 10 ng/mL IL-6, 100 Units/mL penicillin, 20 ng/mL SCF, and 100 µg/mL streptomycin.

### Cloning and purification of recombinant human R1 and R2

Cloning and purification of recombinant human R1 and R2 were performed as described previously^46^. Briefly, the cDNAs of human *RRM1* (Accession NM_001033) and *RRM2* (Accession NM_001034) were subcloned into the expression vector and expressed in *Escherichia coli* cells. 6× His-tagged recombinant proteins were purified with a nickel-nitrilotriacetic acid affinity gel and were used for FP binding assay, AlphaLISA binding assay, and RNR assay. 6× His-tagged recombinant R1 (75 − 742 aa) was purified as above and used for cocrystal structure analysis after removal of 6× His-tag using enzymatic digestion.

### Fluorescence polarization binding assay

TAMRA-labeled P7 peptide was obtained from Peptide Institute, Inc. to be used as a fluorescent probe in the FP binding assay. The final reaction mixture was composed of 50 mmol/L Tris-Cl (pH 8.0), 10 mmol/L DTT, 10 nmol/L TAMRA-P7, 32 ng/mL His-R1, and 1% DMSO with or without test compound. The assay was performed for 120 min at room temperature with protection from light, after which FP of each sample was measured with a PHERAstarFS. IC_50_ values were calculated using the SAS software package in EXSUS (CAC Croit).

### Cocrystal structure

The structure of RRM1 complexed with compound 1 was solved as reported previously^46^. Briefly, crystal of the complex was grown using the hanging-drop vapor diffusion method from a reservoir solution containing 10–30% polyethylene glycol 3350, 15% (v/v) glycerol, 50–150 mmol/L Li2SO4, and 100 mmol/L CH3COONa/CH3COOH, pH 6.0, at 25 °C. The program iMOSFLM^57^ from the CCP4 suite^58^ was used to process the data. The space group was P212121. The structure of RRM1 complexed with the inhibitor was solved by molecular replacement method using MOLREP^59^. The search model was based on the human RRM1 structure (Protein Data Bank [PDB] ID: 2WGH^10^). The structure of RRM1 complexed with the inhibitor was refined using REFMAC5^60^. Manual rebuilding of the models and interpretation of the electron density map were performed using COOT^61^. The final model had the following *R* values: *R*_work_ ¼ 18.9% and *R*_free_ ¼ 23.2%. Table 1 summarizes statistics from data collection and refinement.

**Table 1:** Crystallographic data collection and refinement statistics.

	Human RRM1 with compound 1 (PDB ID: 6L3R)
Data collection	
Space group	P 21 21 21
Cell dimensions	
* a*, *b*, *c* (Å)	106.86, 108.40, 130.10
α, β, γ (°)	90.00, 90.00, 90.00
Resolution (Å)	48.34–2.00 (2.11–2.00)
* R*_merge_	0.093 (0.345)
* I* / σ*I*	12.5 (4.8)
Completeness (%)	98.9 (98.5)
Redundancy	5.9 (5.4)
Refinement	
Resolution (Å)	47.92–2.00
No. reflections	96003
* R*_work_/R_free_	18.9/23.2
No. atoms	
Protein	10,511
Ligand/ion	137
Water	323
* B*-factors	
Protein	25.95
Ligand/ion	39.42
Water	22.91
R.m.s. deviations	
Bond lengths (Å)	0.0175
Bond angles (°)	1.7747

^*^Values in parentheses are for highest-resolution shell.

### Surface plasmon resonance analysis

The surface plasmon resonance was measured using Biacore T200 (GE Healthcare). His-R1 was immobilized onto Series S Sensor Chip NTA with Amine Coupling Kit and NTA Reagent Kit (GE Healthcare). The binding of the test compound was measured with a continuous flow of PBS, including 5% DMSO at 30 µL/min at 25 °C. Data analysis, including the calculation of affinity (Kd) value, was performed using Biacore T200 Evaluation Software version 3 (GE Healthcare). The Kd value was determined using steady-state affinity analysis.

### AlphaLISA binding assay

His-R1 purified above and GST-R2 obtained from Abnova Corporation were used as human RNR components, and the AlphaLISA signal was measured to assess the interaction between His-R1 and GST-R2. GST-His (Alpha Diagnostic International Inc.) was used to investigate direct effect of compound on AlphaLISA signal. The final reaction mixture was composed of 50 mmol/L HEPES, 4 mmol/L magnesium acetate, 100 mmol/L KCl, 6 mmol/L DTT, 2 mmol/L ATP, 0.24 mmol/L NADPH, 0.010 mmol/L CDP, 0.1 vol% polysorbate 20, 4.3 μg/mL His-R1, 3.3 μg/mL GST-R2, 20 μg/mL Alpha Glutathione Donor beads (PerkinElmer, Inc.), 20 μg/mL Nickel Chelate AlphaLISA Acceptor beads (PerkinElmer, Inc.), and 1 vol% DMSO with or without test compound. The mixture was incubated for 60 min at room temperature with protection from light, after which the AlphaLISA signal of each sample was measured with an EnSpire® (PerkinElmer, Inc.). The experiment was performed in triplicates. IC_50_ values were calculated using the SAS software package.

### Immunoprecipitation assay

His-R1 purified above and GST-R2 obtained from Abnova Corporation were used as human RNR components. The final reaction mixture was the same as AlphaLISA binding assay. Anti-RRM1 antibody (#ab137114) and Rabbit IgG (#ab172730) were used for immunoprecipitation.

### RNR assay

The inhibition of RNR activity by the test compound was evaluated by measuring the formation of dCDP from CDP, as reported previously^36^. The assay was performed for 30 min at 37 °C in a reaction mixture composed of 50 mmol/L HEPES, 4 mmol/L magnesium acetate, 100 mmol/L KCl, 6 mmol/L DTT, 2 mmol/L ATP, 0.24 mmol/L NADPH, 10.0 µmol/L CDP, 0.1 mg/mL BSA, 8.0 µg/mL His-R1, 2.4 µg/mL His-R2, and 1% DMSO with or without test compound. The reaction was discontinued by heat treatment at 95 °C for 5 min. The amount of dCDP after the reaction was quantified by a prominence HPLC system and LabSolutions software (SHIMADZU CORPORATION). Separations were performed at 265 nm in a Shim-pack XR-ODS column of particle size 2.2 μm (100 × 3.0 mm, SHIMADZU GLC Ltd.). Overall, 5 µL of each sample was injected, and chromatography was performed at 40 °C. Mobile A was composed of 10 mmol/L potassium dihydrogenphosphate, 10 mmol/L tetrabutylammonium hydroxide, 0.25% methanol and 8 mmol/L HCl. Mobile B was composed of 50 mmol/L potassium dihydrogenphosphate, 5.6 mmol/L tetrabutylammonium hydroxide, and 30% methanol. The gradient was as follows: 52–60% B for 9 min, 60–99% B for 1 min, 99% B for 5 min, 99–52% B for 1 min, and 52% B for 5 min. The flow rate was 0.5 mL/min. The experiment was performed in triplicates. IC_50_ values were calculated using the SAS software package.

### Cell proliferation assay

Cell proliferation assay was performed as reported previously^62^. Briefly, cells were plated and incubated for 1 day, followed by 3 days of exposure to the compound. After compound exposure, viable cells were detected by using the CellTiter-Glo luminescent cell viability assay (Promega). GI_50_ values were calculated using the SAS software package. The experiment was performed in triplicates.

### Fluoppi assay

The cDNA of human *RRM1* (75–742 aa) and human *RRM2* was subcloned into the expression vectors phAG-MNL and pAsh-MCL (MEDICAL & BIOLOGICAL LABORATORIES CO., LTD.), respectively. The constructs were co-transfected using ViaFect (Promega) into HCC38 or NCI-H460 cell lines. After overnight incubation, cells were treated with the test compound, followed by fluorescent image analysis by Opera Phenix^TM^ (Perkin Elmer, Inc.).

### Immunoblotting analysis

Immunoblotting analysis was performed as reported previously^62^. The collected cells and tissue samples were homogenized on ice with M-PER and T-PER buffer (Thermo Fisher Scientific, Inc.), respectively. Proteins were separated by SDS-PAGE and analyzed by Western blotting. Anti-Chk1 antibody (#2360), anti-Phospho-Chk1 (Ser345) antibody (#2348), anti-Cleaved PARP antibody (#5625), anti-Cleaved Caspase-3 antibody (#9661), anti-β-Actin antibody (#4967), HRP-conjugated anti-rabbit IgG antibody (#7074), and HRP-conjugated anti-mouse IgG antibody (#7076) were obtained from Cell Signaling Technology, Inc. Anti-phospho-RPA2 (Thr21) antibody (#ab61065) and anti-RRM1 antibody (#ab137114) were obtained from Abcam plc. Anti-SLFN11 antibody (#sc515071) and anti-RRM2 antibody (#sc10844) were obtained from Santa Cruz Biotechnology, Inc. Anti-RPA2 antibody (#NA19L), anti-phospho-RPA2 (Ser4/Ser8) antibody (#A300-245A), and anti-γH2AX antibody (#613402) were obtained from Merck KGaA, Bethyl Laboratories, Inc., and BioLegend, Inc., respectively. The signals were detected with the ImageQuant LAS 4010 (GE Healthcare UK Ltd.).

### Immunofluorescence staining

Cells were seeded onto 8-well chamber slides and incubated for 1 day, followed by compound treatment for 2 h. The cells were fixed with 4% paraformaldehyde phosphate buffer and permeabilized with PBS, including 0.5% Triton(R) X-100. Permeabilized cells were incubated with the primary antibody for anti-phospho-RPA2 (Thr21) (#ab61065, Abcam), followed by Alexa 594-labeled secondary antibody (#A-11037, Thermo Fisher Scientific Inc.), and mounted in Vectashield HardSet Mounting Medium with DAPI (Vector Laboratories Inc.). Slides were examined using a confocal laser scanning microscope LSM800 (Carl Zeiss, Inc.).

### Correlation analysis

Correlation analysis of *SLFN11* expression and TAS1553 cytotoxicity was performed using Pearson’s correlation coefficient (P < 0.05) in JMP 13 software. The gene expression data of cell lines were available online CCLE (http://www.broadinstitute.org/ccle/home). TAS1553 cytotoxicity data from 22 cell lines except BHK-89, Ca9-22, and DOK in Supplementary Table 2 were used for correlation analysis because the gene expression data on CCLE are not present for the three cell lines.

### siRNA treatment

A673 cells were transfected with 1 nM siRNAs againt SLFN11 or 0.1 nM siRNAs against RRM1, and NCI-H460 cells were transfected with 0.01 nM siRNAs against RRM1 for 24 h using Lipofectamine^®^ RNAiMAX Reagent (Thermo Fisher Scientific Inc.) and then re-seeded onto a 96-well plate for cell proliferation assay and caspase induction assay and onto dishes for immunoblotting analysis. siRNAs were obtained from Thermo Fisher Scientific Inc. as follows: Silencer® Select Negative Control #1 siRNA (#4390843 used as siControl), SLFN11 Silencer® Select Pre-designed siRNA (#s40703 used as siSLFN11-1 and #s40704 as siSLFN11-2), RRM1 Silencer® Select Pre-designed siRNA (#s12357 used as siRRM1-1 and #s12359 as siRRM1-2).

### Caspase induction analysis

Caspase induction analysis was performed as reported previously^62^. Briefly, the cells were seeded, incubated for 1 day, and then treated with compound exposure for 24 h. Caspase induction was detected by using the Caspase-Glo 3/7 assay (Promega). Relative caspase-3/7 activation was calculated by applying the correction with respect to cell number. The experiment was performed in triplicates.

### Antitumor activity experiments

Animal protocols were approved according to regional Institutional Animal Care and Use Committees. Female 4-weeks-old F344/NJcl-rnu/rnu rats and male 5-weeks-old BALB/cAJcl-nu/nu mice were obtained from CLEA Japan, male 5-weeks-old B6N-Tyrc-Brd/BrdCrCrl were obtained from Charles River Laboratories.

For the subcutaneous implantation model of MV-4-11, F344/NJcl-rnu/rnu rats were irradiated with X-ray (4 Gy) to improve the efficiency of xenograft implantation. Two days after X-ray irradiation, MV-4-11 cells suspended in PBS with 50 vol% Matrigel were inoculated into the rats (2 × 10^7^ cells/animal). In the mice subcutaneous implantation model, HCC38 cells suspended same as above were inoculated into male BALB/cAJcl-nu/nu mice (1 × 10^7^ cells /body). The animals were randomized and administered with the compound for 14 days. Antitumor activity and body weight change were evaluated as previously reported^62^. Dunnett test using the SAS software package in EXSUS (CAC Croit) was used to compare the tumor volume (*P* < 0.05).

For the systemic AML model, B6N-Tyr^c-Brd^/BrdCrCrl mice were irradiated with X-ray (6 Gy) to improve the efficiency of implantation. One day after X-ray irradiation, cells harboring the human *MLL-AF9* fusion gene and *Luciferase* established from mice bone marrow were suspended in PBS and inoculated into the mice via the tail vein (1 × 10^7^ cells/body). Eight days after inoculation, animals were randomized to the test groups based on the photon values as reported previously^62^. After 3 days of grouping, the administration with the test compound was started. An increase in life span (ILS) was determined with the following formula: ILS (%) = ([median survival time (MST) of the treated group]/[MST of control] – 1) × 100. Log-Lank test using the SAS software package was used to compare the MST (*P* < 0.05).

TAS1553 and capecitabine were formulated in 0.5% hypromellose solution. Cytarabine was formulated in saline, and paclitaxel was formulated in saline, including 5% ethanol and 5% cremophore EL.

### Measurement of TAS1553 in plasma

F344/NJcl-rnu/rnu rats were orally administered with TAS1553 (two animals per group). The plasma samples were collected at each point. TAS1553 concentrations in plasma were measured with the LC-MS/MS.

### Measurement of intracellular metabolites

Cells were plated, cultured for 1 day, treated with the compound, and harvested. The collected cells were homogenized with 0.42 mol/L perchloric acid, including IS-dATP at 0.6 μmol/L and IS-ATP at 2.4 μmol/L as internal standards for dATP and ATP, respectively. At each time point, xenograft tumors were excised after single dosing of the test compound. The tissues were homogenized with 0.42 mol/L perchloric acid, including IS-dATP at 3 μmol/L as an internal standard for dATP and ATP. Subsequently, a dichloromethane solution containing 0.5 N tri-n-octylamine was added to the perchloric acid-soluble fraction obtained by centrifugation. The aqueous layer collected after vortex and centrifugation was analyzed by LC-MS/MS. LC-MS/MS analysis was performed with a prominence HPLC system connected to a LCMS-8040 tandem mass spectrometer with ESI probe (SHIMADZU CORPORATION). Chromatographic separations were performed on InertSustainBio C18 HP of particle size 3 μm (100 × 2.1 mm, GL Science Inc.). Overall, 5 µL of each sample was injected, and chromatography was performed at 40 °C. Mobile A was 5 mmol/L dibutylammonium acetate solution. Mobile B was methanol. The gradient was as follows: 1–80% B for 3 min, 80% B for 4.2 min, 80-1% B for 0.1 min, and 1% B for 4.7 min. The flow rate was 0.2 mL/min. Nitrogen was used as the nebulizer gas and reached 3.00 L/min. Argon was used as the collision-induced dissociation gas and reached 15.00 L/min. Desolvation line temperature and heat block temperatures were 250 °C and 400 °C, respectively. The multiple reaction monitoring (MRM) transitions are summarized in Supplementary Table 4. Dunnett test was performed using the SAS software package in EXSUS (CAC Croit) to compare the intracellular or intratumoral dATP pool (*P* < 0.05).

### Statistics and reproducibility

Statistical analysis was performed using the SAS software package in EXSUS (CAC Croit). IC_50_ values for each assay were also calculated using the SAS software package in EXSUS (CAC Croit). IC_50_ values for FP binding assay, AlphaLISA binding assay and RNR assay were determined in three independent experiments performed in triplicate and IC_50_ values for cell profileration were determined in the experiment performed in triplicate. For comparison between two groups, Student’s *t*-test was applied. Multiple comparisons were performed with using Dunnett test. Log-rank test was used for comparison of survival curve. Correlation analysis was performed using Peason’s correlation coefficient in JMP13 software. *P* < 0.05 was considered statistically significant. Figures 1e, 2a, e–g, and 3c–e and Supplementary Figs. 2, 4, 7, and 8 are representative of two to three independent experiments.

### Reporting summary

Further information on research design is available in the Nature Research Reporting Summary linked to this article.

## Supplementary information

Supplementary Information

Reporting Summary

## Data Availability

Presented X-ray co-crystal structure is available as 6L3R on the Protein Data Bank. Uncropped versions of western blots and individual tumor growth curves are provided as Supplementary Figs. 9 and 10, respectively. All data are available online or from the corresponding authors upon reasonable request.
