# Supplementary material for: TAS1553, a small molecule subunit interaction inhibitor of ribonucleotide reductase, exhibits antitumor activity by causing DNA replication stress
Source: Commun Biol. 2022 Jun 9;5:571. doi: 10.1038/s42003-022-03516-4 (PMC9184620; doi:10.1038/s42003-022-03516-4)
Supplement: Supplementary file 1 — Supplementary Information [file 42003_2022_3516_MOESM1_ESM.pdf]

Supplementary Table 1

	Assay Name	Species	Concentration	% inibition
1	Adenosine A_1	hum	10 μmol/L	7
2	Adenosine A_{2A}	hum	10 μmol/L	8
3	Adenosine A_3	hum	10 μmol/L	-4
4	Adrenergic α_{1A}	rat	10 μmol/L	9
5	Adrenergic α_{1B}	rat	10 μmol/L	3
6	Adrenergic α_{1D}	hum	10 μmol/L	4
7	Adrenergic α_{2A}	hum	10 μmol/L	1
8	Adrenergic β_1	hum	10 μmol/L	1
9	Adrenergic β_2	hum	10 μmol/L	4
10	Androgen (Testosterone)	hum	10 μmol/L	19
11	Bradykinin B_1	hum	10 μmol/L	1
12	Bradykinin B_2	hum	10 μmol/L	3
13	Calcium Channel L-Type, Benzothiazepine	rat	10 μmol/L	2
14	Calcium Channel L-Type, Dihydropyridine	rat	10 μmol/L	0
15	Calcium Channel N-Type	rat	10 μmol/L	-3
16	Cannabinoid CB_1	hum	10 μmol/L	10
17	Dopamine D_1	hum	10 μmol/L	9
18	Dopamine D_{2S}	hum	10 μmol/L	16
19	Dopamine D_3	hum	10 μmol/L	5
20	Dopamine $D_{4.2}$	hum	10 μmol/L	7
21	Endothelin ET_A	hum	10 μmol/L	4
22	Endothelin ET_B	hum	10 μmol/L	-3
23	Epidermal Growth Factor (EGF)	hum	10 μmol/L	-2
24	Estrogen ERα	hum	10 μmol/L	-1
25	$GABA_A$, Flunitrazepam, Central	rat	10 μmol/L	11
26	$GABA_A$, Muscimol, Central	rat	10 μmol/L	7
27	$GABA_{B1A}$	hum	10 μmol/L	8
28	Glucocorticoid	hum	10 μmol/L	5
29	Glutamate, Kainate	rat	10 μmol/L	13
30	Glutamate, NMDA, Agonism	rat	10 μmol/L	9
31	Glutamate, NMDA, Glycine	rat	10 μmol/L	12
32	Glutamate, NMDA, Phencyclidine	rat	10 μmol/L	-4
33	Histamine H_1	hum	10 μmol/L	2
34	Histamine H_2	hum	10 μmol/L	-6

	Assay Name	Species	Concentration	% inibition
35	Histamine H_3	hum	10 μmol/L	12
36	Imidazoline I_2, Central	rat	10 μmol/L	10
37	Interleukin IL-1 R1	hum	10 μmol/L	-4
38	Leukotriene, Cysteinyl $CysLT_1$	hum	10 μmol/L	7
39	Melatonin MT_1	hum	10 μmol/L	12
40	Muscarinic M_1	hum	10 μmol/L	4
41	Muscarinic M_2	hum	10 μmol/L	0
42	Muscarinic M_3	hum	10 μmol/L	0
43	Neuropeptide Y Y_1	hum	10 μmol/L	-2
44	Neuropeptide Y Y_2	hum	10 μmol/L	17
45	Nicotinic Acetylcholine	hum	10 μmol/L	-5
46	Nicotinic Acetylcholine α1, Bungarotoxin	hum	10 μmol/L	8
47	Opiate δ_1 (OP1, DOP)	hum	10 μmol/L	6
48	Opiate κ (OP2, KOP)	hum	10 μmol/L	-8
49	Opiate μ (OP3, MOP)	hum	10 μmol/L	11
50	Phorbol Ester	mouse	10 μmol/L	3
51	Platelet Activating Factor (PAF)	hum	10 μmol/L	14
52	Potassium Channel [K_{ATP}]	ham	10 μmol/L	15
53	Potassium Channel hERG	hum	10 μmol/L	19
54	Prostanoid EP_4	hum	10 μmol/L	3
55	Purinergic P2X	rabbit	10 μmol/L	-8
56	Purinergic P2Y	rat	10 μmol/L	3
57	Rolipram	rat	10 μmol/L	4
58	Serotonin (5-Hydroxytryptamine) $5\text{-}HT_{1A}$	hum	10 μmol/L	0
59	Serotonin (5-Hydroxytryptamine) $5\text{-}HT_{2B}$	hum	10 μmol/L	2
60	Serotonin (5-Hydroxytryptamine) $5\text{-}HT_3$	hum	10 μmol/L	3
61	Sigma σ_1	hum	10 μmol/L	9
62	Sodium Channel, Site 2	rat	10 μmol/L	-4
63	Tachykinin NK_1	hum	10 μmol/L	-4
64	Thyroid Hormone	rat	10 μmol/L	7
65	Transporter, Dopamine (DAT)	hum	10 μmol/L	-8
66	Transporter, GABA	rat	10 μmol/L	10
67	Transporter, Norepinephrine (NET)	hum	10 μmol/L	-4
68	Transporter, Serotonin (5-Hydroxytryptamine) (SERT)	hum	10 μmol/L	5

Supplementary Table 1 Evaluation of TAS1553 in a Eurofins LeadProfilingScreen panel assay.

Supplementary Table 2

Cell line	Origin	TAS1553 GI_{50} (μmol/L)	HU GI_{50} (μmol/L)
THP-1	Acute monocytic leukemia	0.894	40.8
HEL	Acute myelogenous leukemia	1.58	112
HL-60	Acute promyelocytic leukemia	2.12	131
K-562	Chronic myelogenous leukemia	2.35	506
BHL-89	Chronic lymphocytic leukemia	4.15	384
MV-4-11	Biphenotypic B myelomonocytic leukemia	0.393	10.8
RPMI8226	Multiple myeloma	0.547	47.1
HCC1599	Breast cancer (Basal type)	0.850	549
HCC1806	Breast cancer (Basal type)	0.228	240
HCC38	Breast cancer (Basal type)	0.352	60.0
MCF-7	Breast cancer (Luminal type)	0.794	518
A549	Non-small cell lung cancer (adenocarcinoma)	1.60	164
NCI-H460	Non-small cell lung cancer (large cell carcinoma)	0.908	192
NCI-H2170	Non-small cell lung cancer (squamous cell carcinoma)	1.20	192
HCT116	Colorectal cancer	0.847	406
NUGC-3	Gastric cancer	1.59	417
CFPAC-1	Pancreatic cancer	0.642	165
786-O	Kidney cancer	1.20	850
DU145	Prostate cancer	0.642	206
A2780	Ovarian cancer	0.856	161
MSTO-211H	Mesothelioma	0.625	>1000
COLO 792	Melanoma	2.08	339
Ca9-22	Gingival cancer	0.955	329
DOK	Displastic keratinocyte	1.82	152
A673	Ewing sarcoma	1.12	42.5

Supplementary Table 2 Inhibitory activity of TAS1553 and HU against cell proliferation in solid and hematopoietic human cancer cell lines. Cells were plated and incubated for 1 day, followed by 3 days of exposure to the indicated compounds. GI_{50} is determined from experiments in triplicate.

Supplementary Table 3

Dose	25 mg/kg	50 mg/kg	100 mg/kg	200 mg/kg
AUC_{0-24} (μmol/L*h)	11.89	27.17	60.64	109.45
C_{max} (μmol/L)	8.37	19.59	38.08	60.29
T_{max} (h)	0.25	0.25	0.25	0.25

Supplementary Table 3 Pharmacokinetic analysis of TAS1553. The plasma samples were collected at 0.25-, 0.5-, 1-, 2-, 4-, 6-, 8-, and 24-hour post-administration from two nude rats at each sampling point per group.

Supplementary Table 4

	Precursor ion (m/z)		Product ion (m/z)	Q1 Pre Bias (V)	CE	Q3 Pre Bias (V)
dATP	490.00	→	159.10	18.0	30.0	29.0
dCTP	466.00	→	159.10	17.0	26.0	29.0
dTTP	481.00	→	159.10	17.0	26.0	30.0
ATP	506.00	→	272.95	28.0	30.0	29.0
IS-dATP	495.05	→	159.05	18.0	31.0	28.0
IS-ATP	514.20	→	159.00	20.0	32.0	27.0

Supplementary Table 4 The multiple reaction monitoring (MRM) transitions for measurement of intracellular metabolites

Supplementary Fig. 1

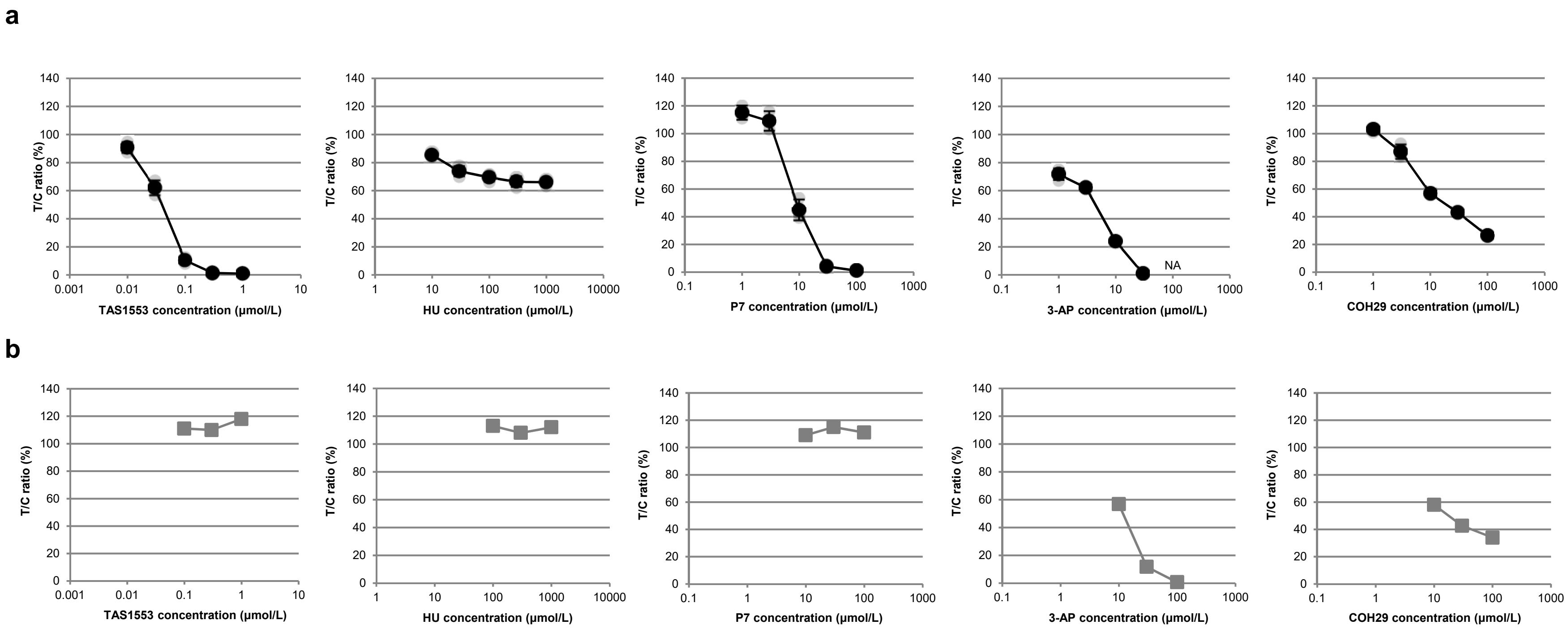

Supplementary Fig. 1 Effect of TAS1553 on RNR subunit interaction. a Concentration-dependent effect of compounds on the protein-protein interaction between human R1 and R2, as measured by the AlphaLISA binding assay. Data are presented as mean ± SD obtained from 3 independent experiments performed in triplicate. **b** Concentration-dependent effect of compounds on AlphaLISA signal. NA, not applicable**.**

Supplementary Fig. 2

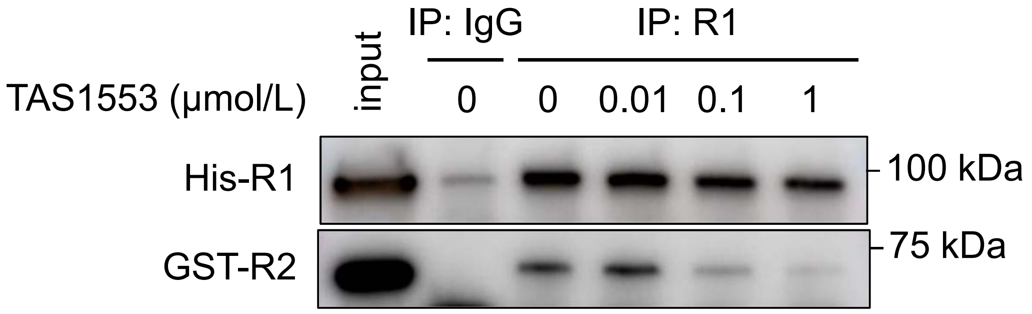

Supplementary Fig. 2 Effect of TAS1553 on interaction between R1 and R2 in co-immunoprecipitation assay. Recombinant His-R1 were mixed with GST-R2 in RNR enzyme assay buffer in the presence of TAS1553, immunoprecipitated with Anti-RRM1 antibody, and analyzed by immunoblotting.

Supplementary Fig. 3

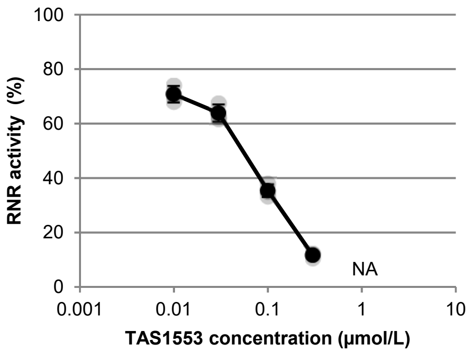

Supplementary Fig. 3 Effect of TAS1553 on RNR enzymatic activity. Concentration-dependent effect of TAS1553 on RNR activity. Enzymatic activity of RNR composed of human R1 and R2 was determined by measuring the production of dCDP from CDP. NA, not applicable**.**

Supplementary Fig. 4

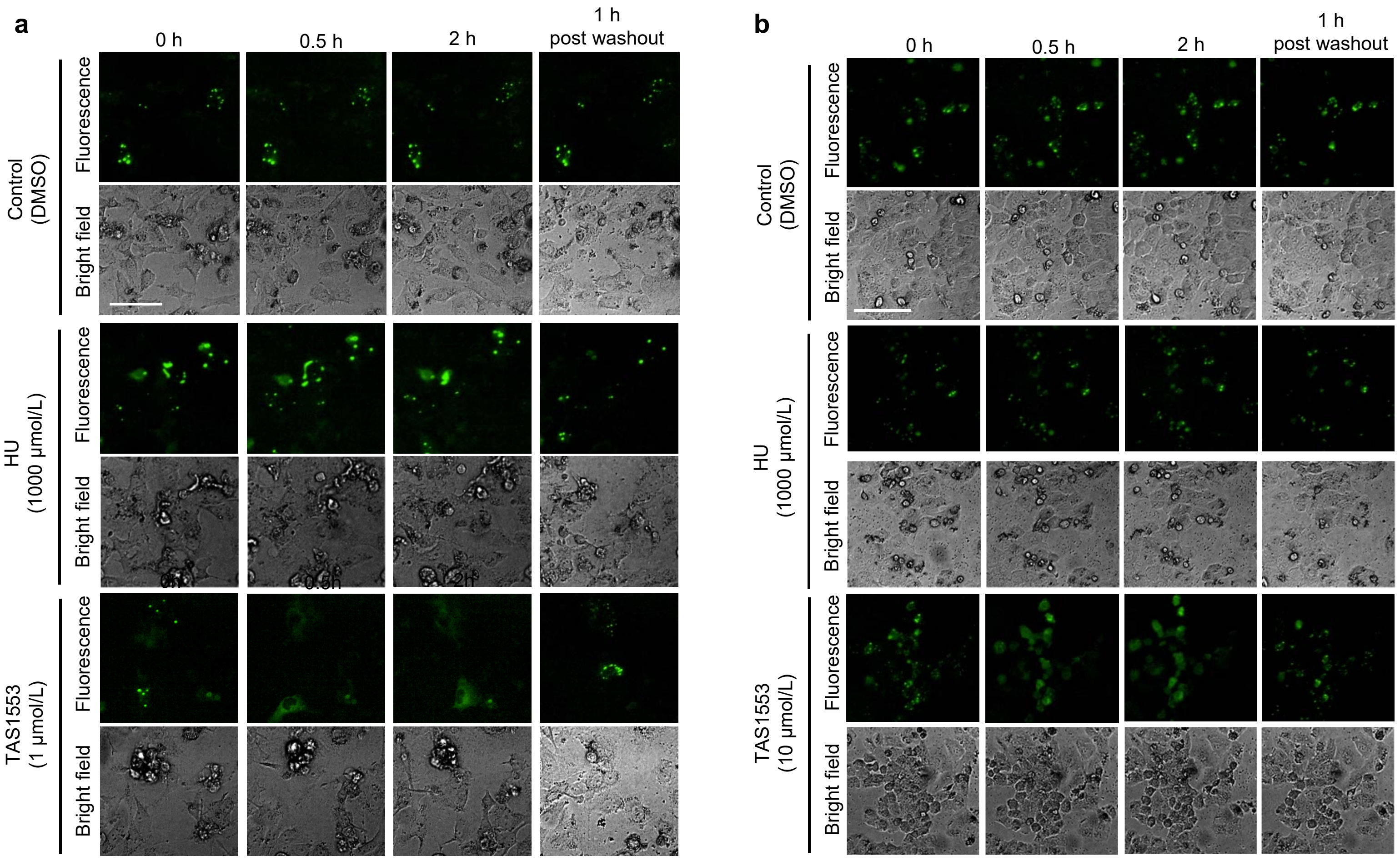

Supplementary Fig. 4 Effect of TAS1553 and HU on intracellular protein-protein interaction between R1 and R2. HCC38 (**a**) or H460 (**b**) cells were co-transfected with cDNAs encoding AG-tagged R1 and Ash-tagged R2, and treated with each compound for 2 h, followed by incubated with drug-free medium for 1 h. Fluorescence and bright-field images were obtained at the indicated hours after compound treatment. Interaction events are visible as green dots in fluorescence images. Images of cells treated with DMSO as control, TAS1553 at 1 or 10 µmol/L, and HU at 1000 µmol/L. Scale bar, 100 µm.

Supplementary Fig. 5

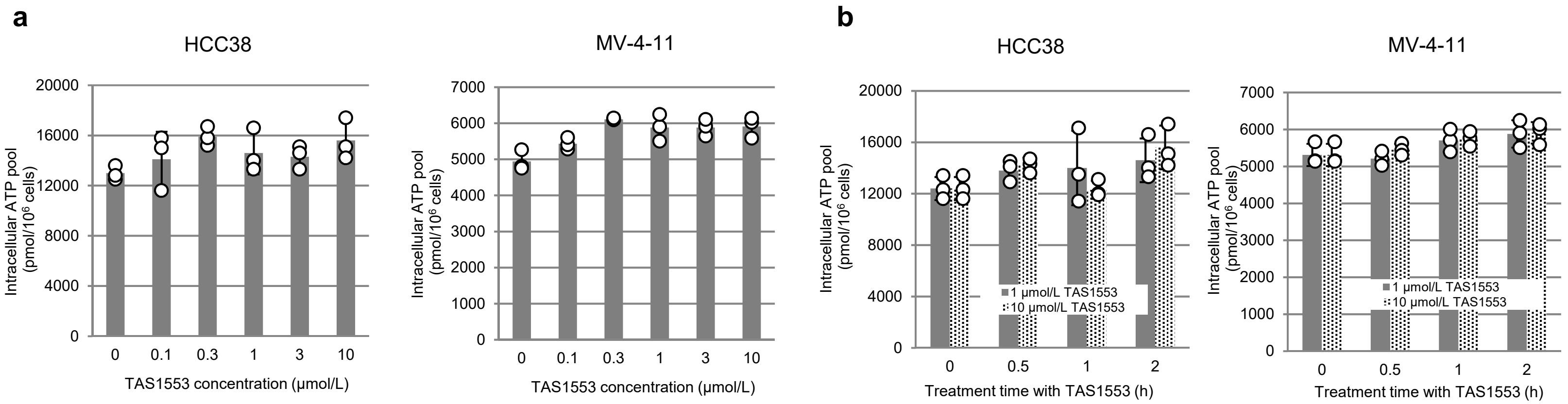

Supplementary Fig. 5 Effect of TAS1553 on intracellular ATP pool in HCC38 and MV-4-11 cells. a, Concentration-dependent changes of the ATP pool in HCC38 cells and MV-4-11 cells treated with TAS1553 for 2 h. **b**, Time-dependent changes of the ATP pool in HCC38 cells and MV-4-11 cells treated with TAS1553. Data are presented as the mean ± SD (N=3).

Supplementary Fig. 6

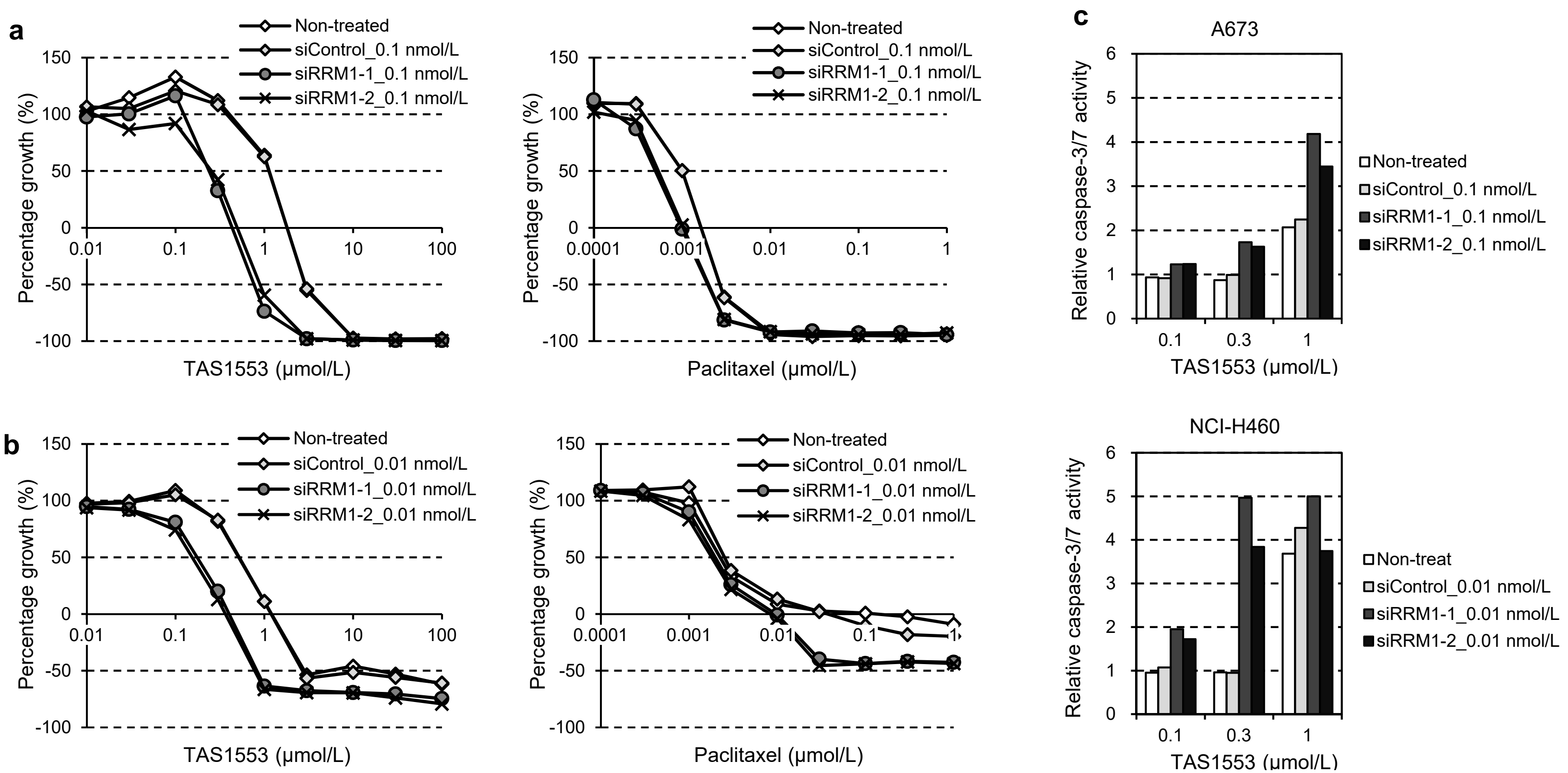

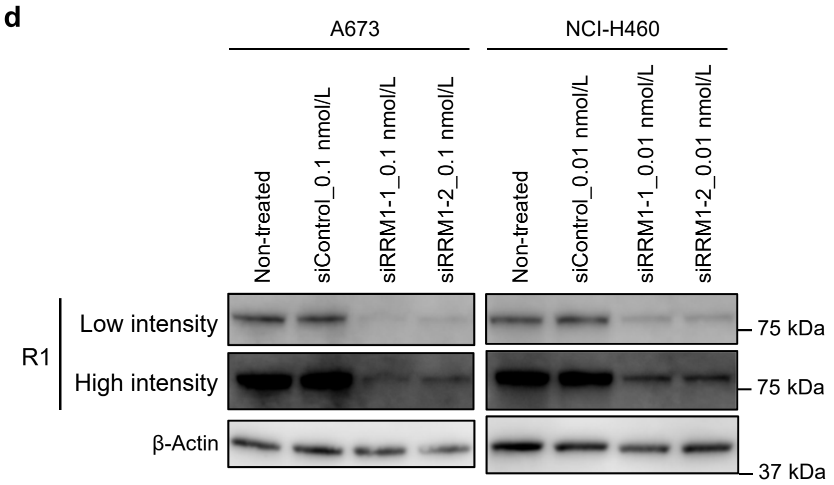

Supplementary Fig. 6 Effect of R1 knockdown on growth inhibition and caspase activation caused by TAS1553. **a,b**, Effects of TAS1553 and paclitaxel on the proliferation of A673 cells (**a**) and NCI-H460 cells (**b**) treated with siRNA against R1 subunit. The cells were treated with the test compound for 3 days after treatment with each siRNA. **c**, Caspase3/7 activation by TAS1553 in A673 cells and NCI-H460 cells treated with siRNA against R1. **d**, R1 expression in A673 cells and NCI-H460 cells treated with siRNA. The cells were treated with siRNA for 24h, cultured for 24h, and then harvested and analyzed by immunoblotting.

Supplementary Fig. 7

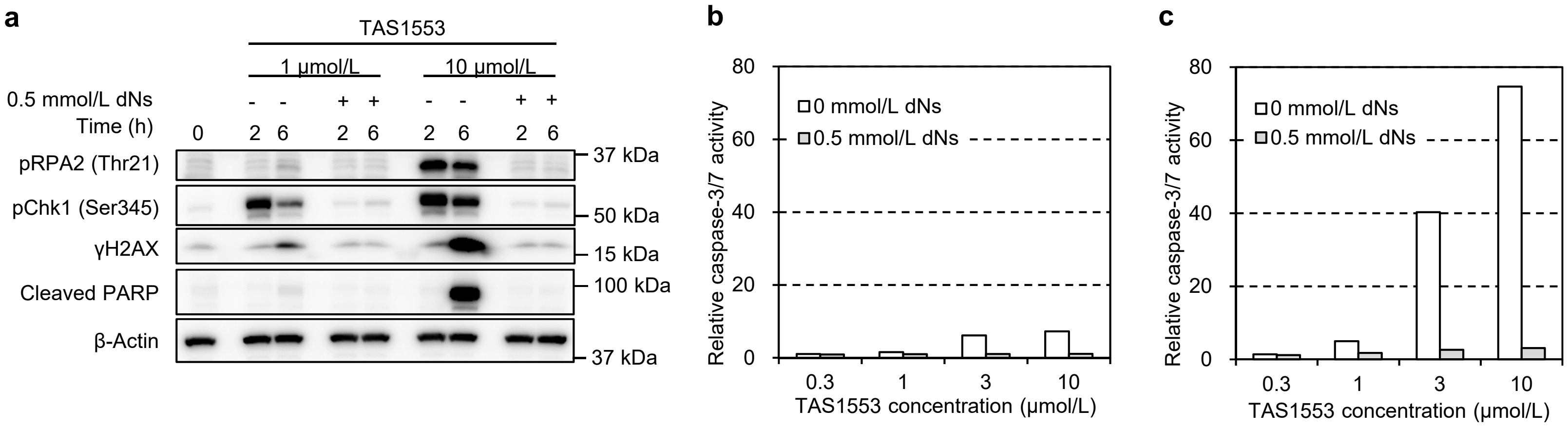

Supplementary Fig. 7 Effect of nucleosides addition on DNA replication and apoptosis caused by TAS1553. MV-4-11 cells were treated with TAS1553 in the presence or absence of 4 deoxyribonucleosides (dAdo, dThd, dGuo, dCyd), harvested at indicated time points, and analyzed by immunoblotting (**a**) and Caspase induction assay after 6 h (**b**) or 24 h (**c**) treatment.

Supplementary Fig. 8

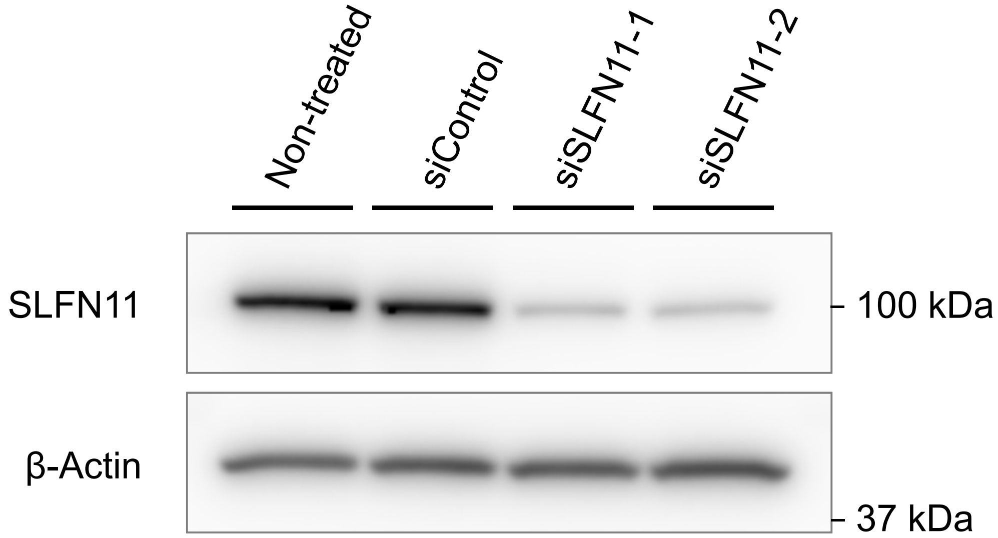

Supplementary Fig. 8 SLFN11 expression in A673 cells treated with siRNA. A673 cells were treated with siRNA against SLFN11 for 24 h, and then harvested and analyzed by immunoblotting.

Supplementary Fig. 9

Uncropped images of western blots (Fig. 2e)

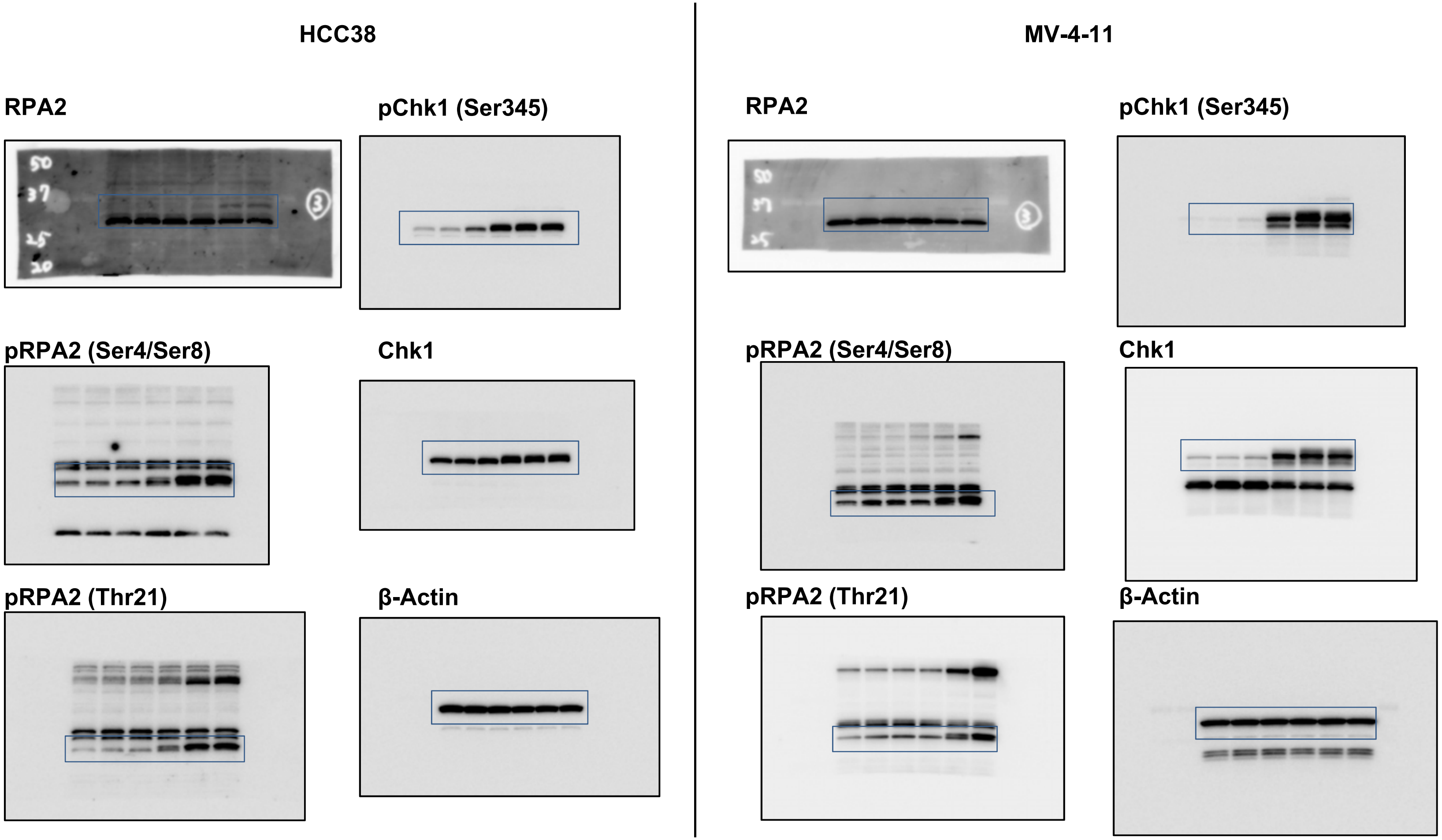

Supplementary Fig. 9

Uncropped images of western blots (Fig. 2g)

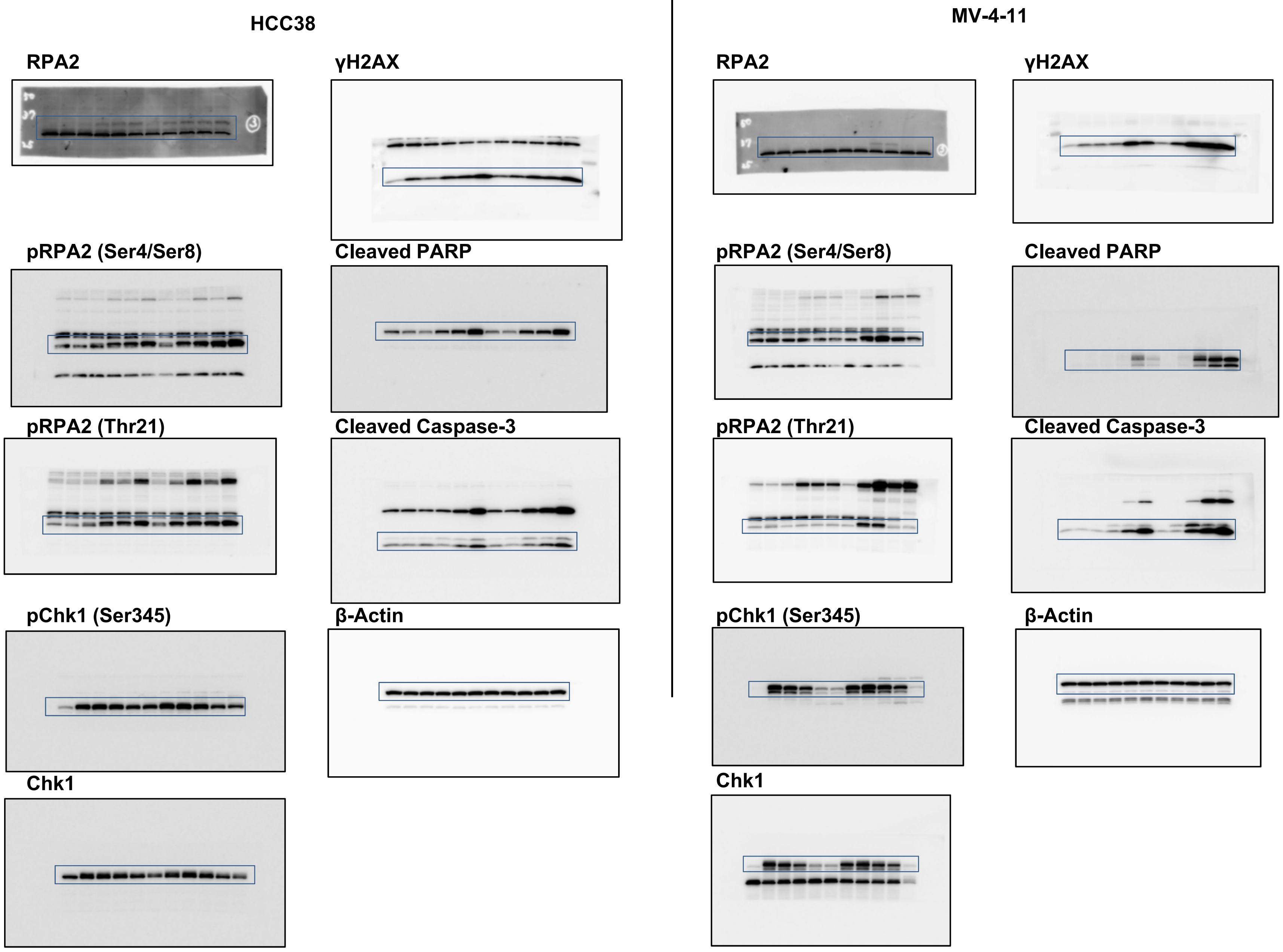

Supplementary Fig. 9

Uncropped images of western blots (Fig. 4d)

pChk1 (Ser345)

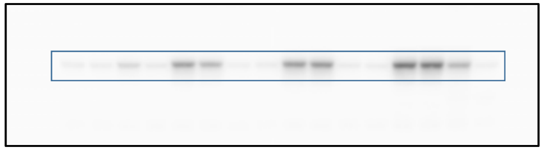

Cleaved PARP

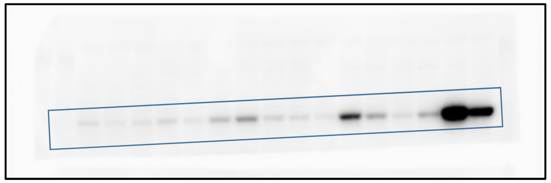

β-Actin

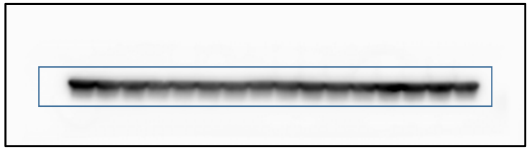

Chk1

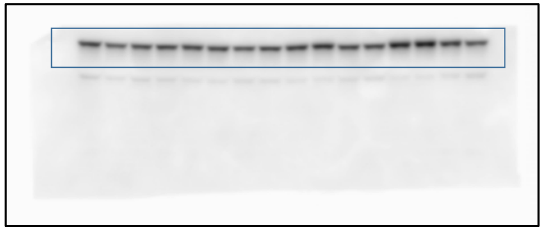

Cleaved Caspase-3

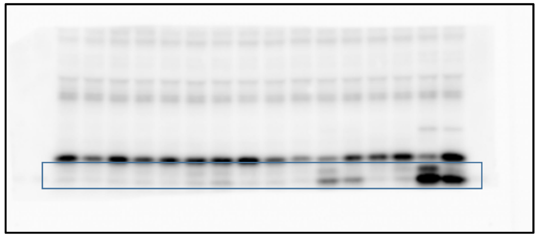

Supplementary Fig. 9

Uncropped images of western blots (Supplementary Fig. 2)

His-R1

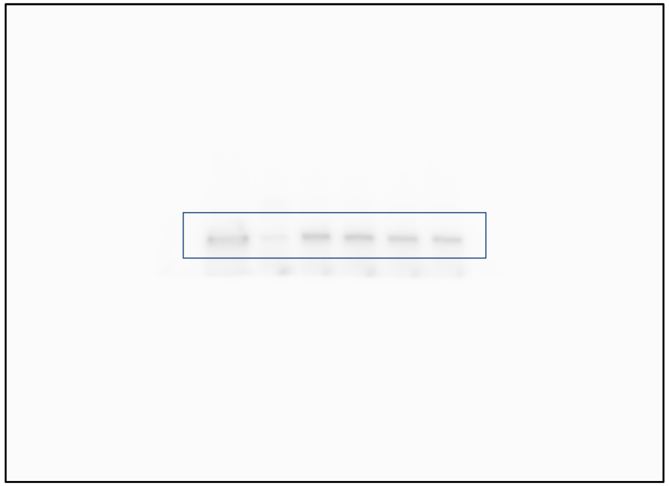

GST-R2

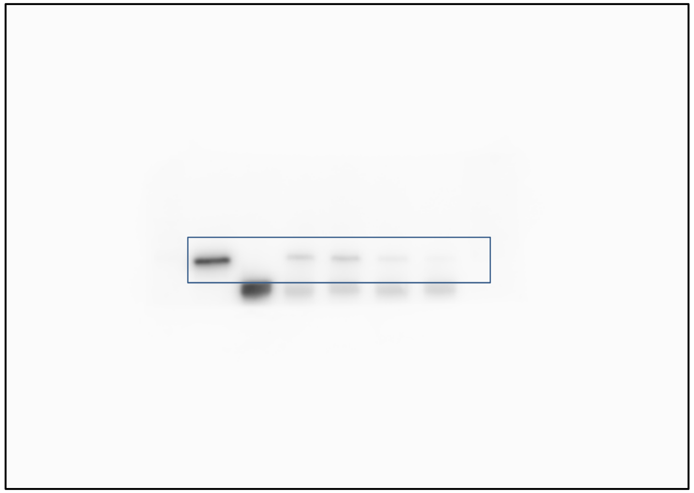

Supplementary Fig. 9

Uncropped images of western blots (Supplementary Fig. 6)

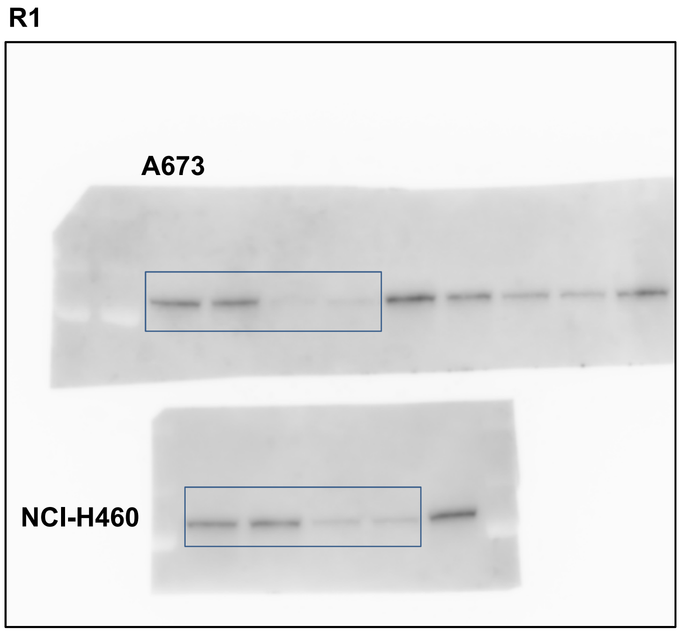

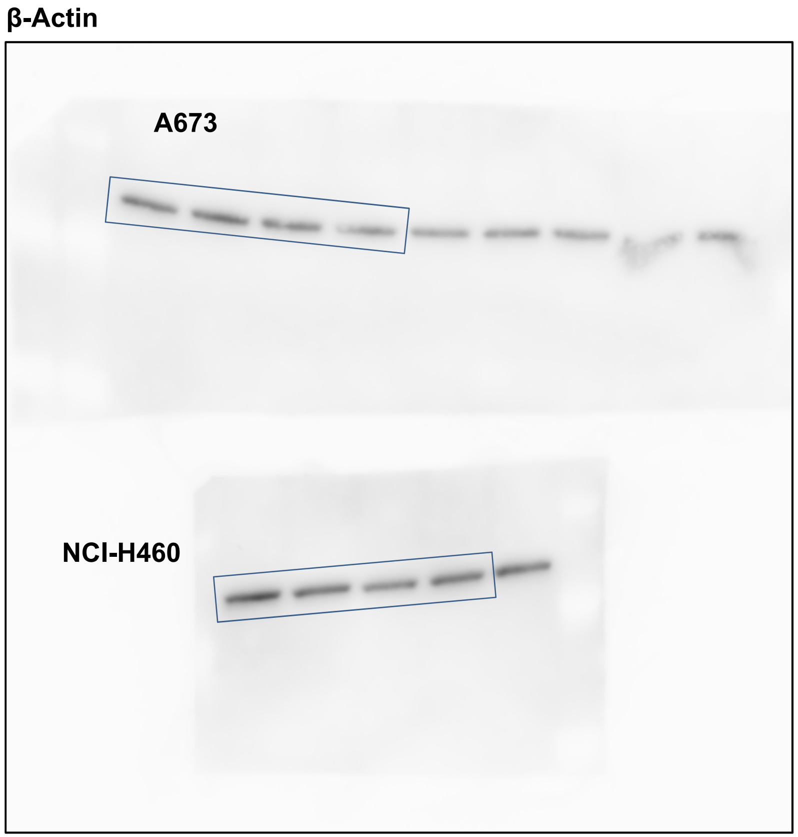

Supplementary Fig. 9

Uncropped images of western blots (Supplementary Fig. 7)

pRPA2 (Thr21)

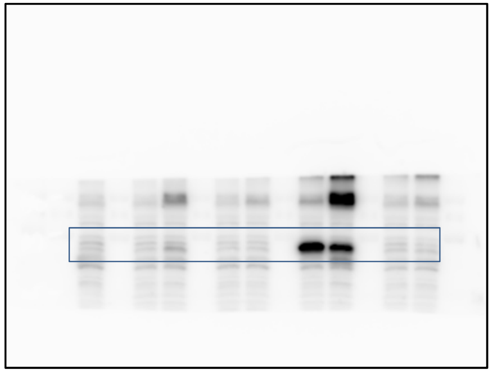

γH2AX

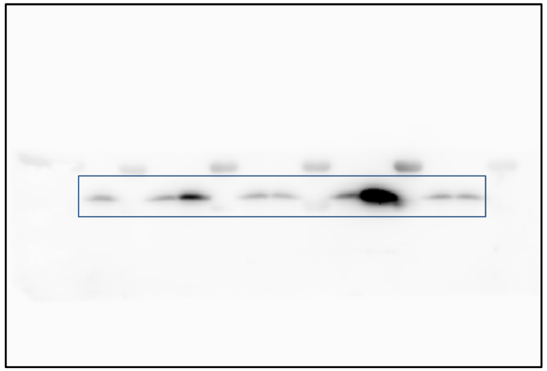

β-Actin

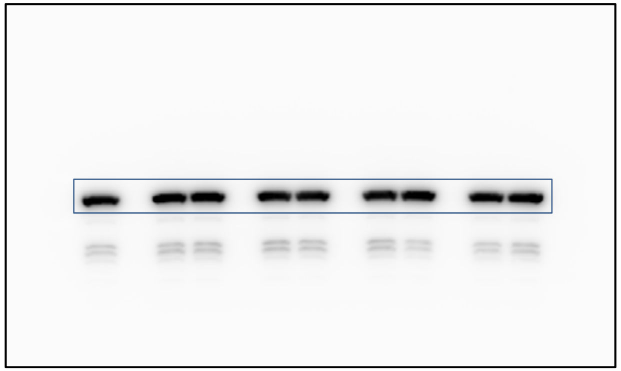

pChk1 (Ser345)

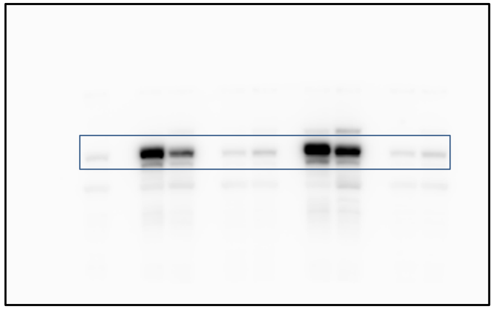

Cleaved PARP

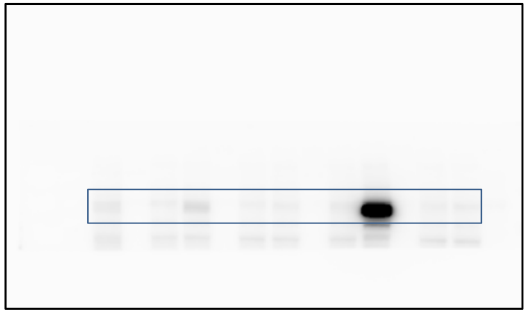

Supplementary Fig. 9

Uncropped images of western blots (Supplementary Fig. 8)

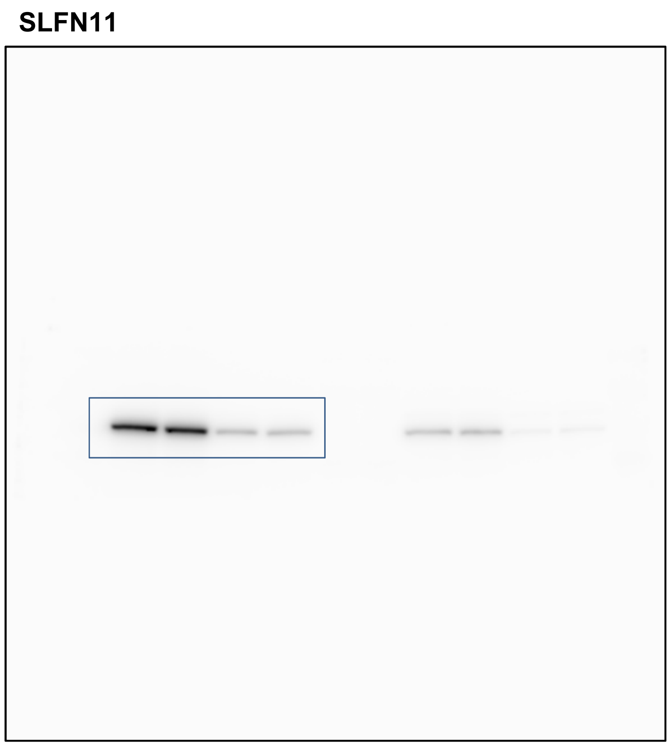

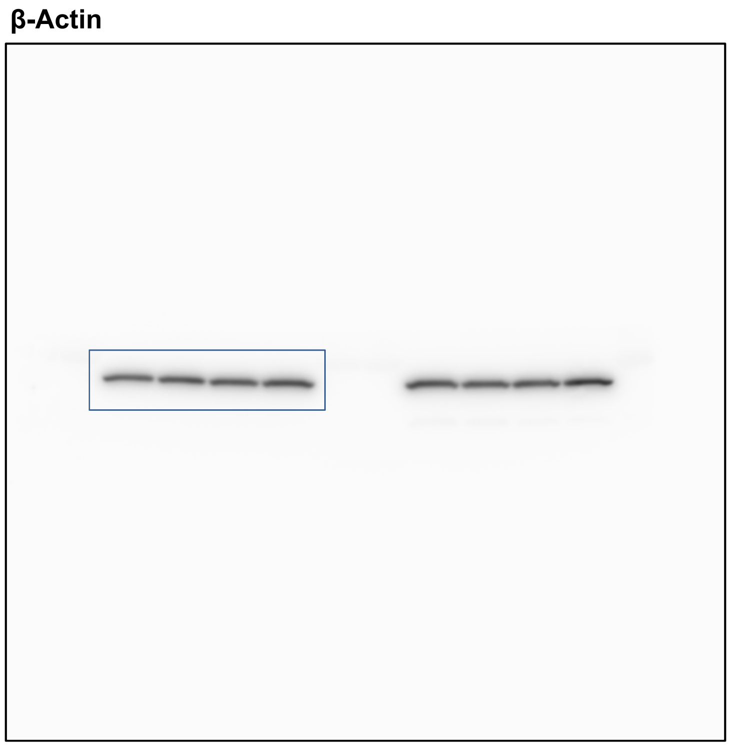

Supplementary Fig. 10

Individual tumor growth curves (Fig. 4e)

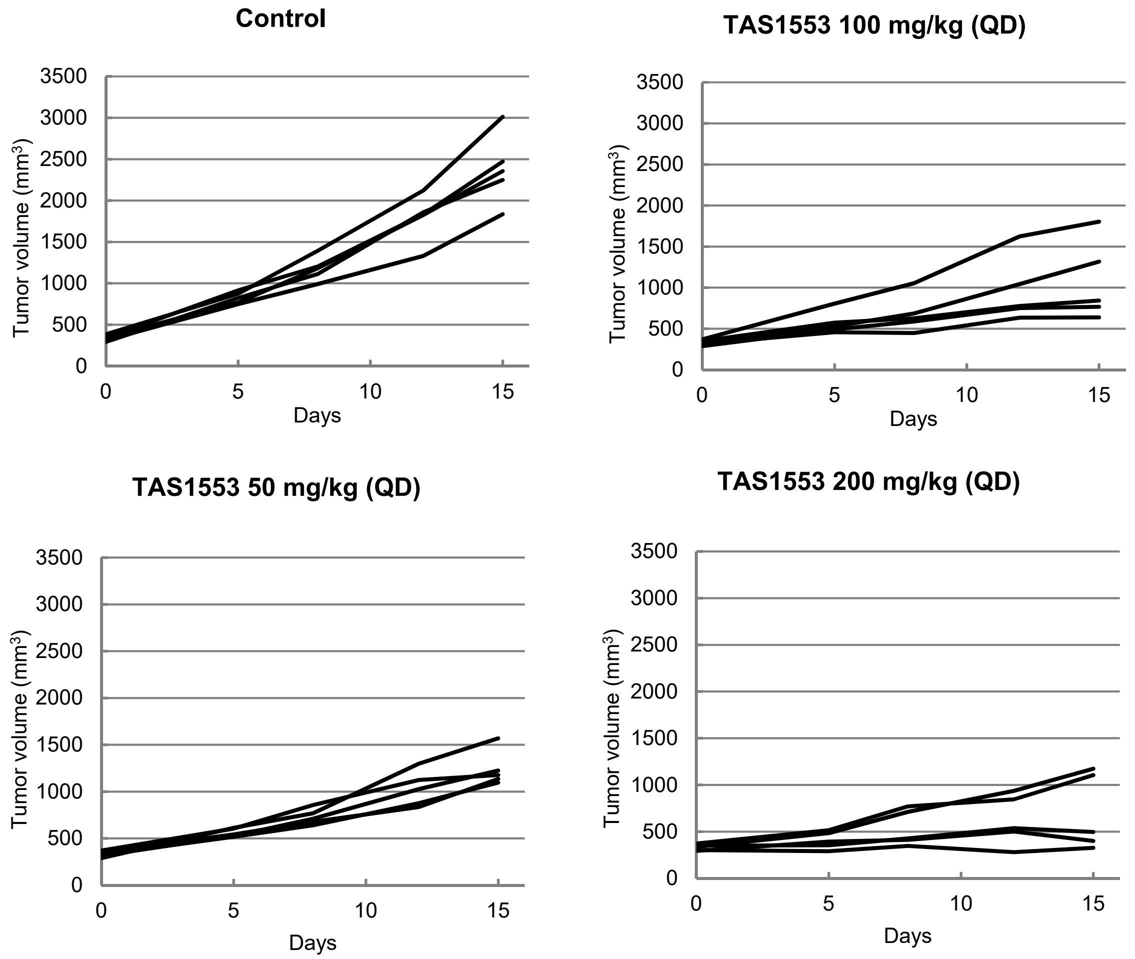

Supplementary Fig. 10

Individual tumor growth curves (Fig. 5a)

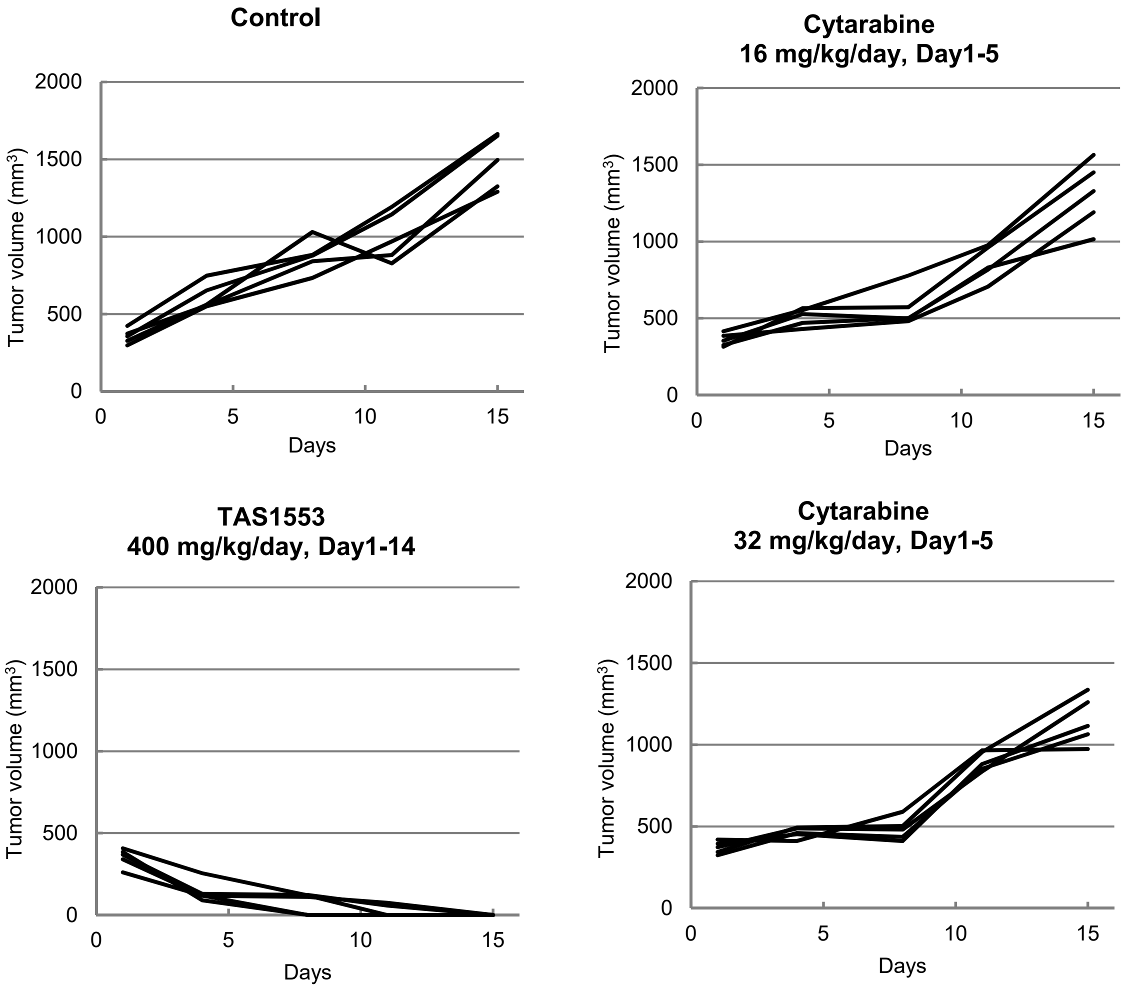

Supplementary Fig. 10

Individual tumor growth curves (Fig. 5c)

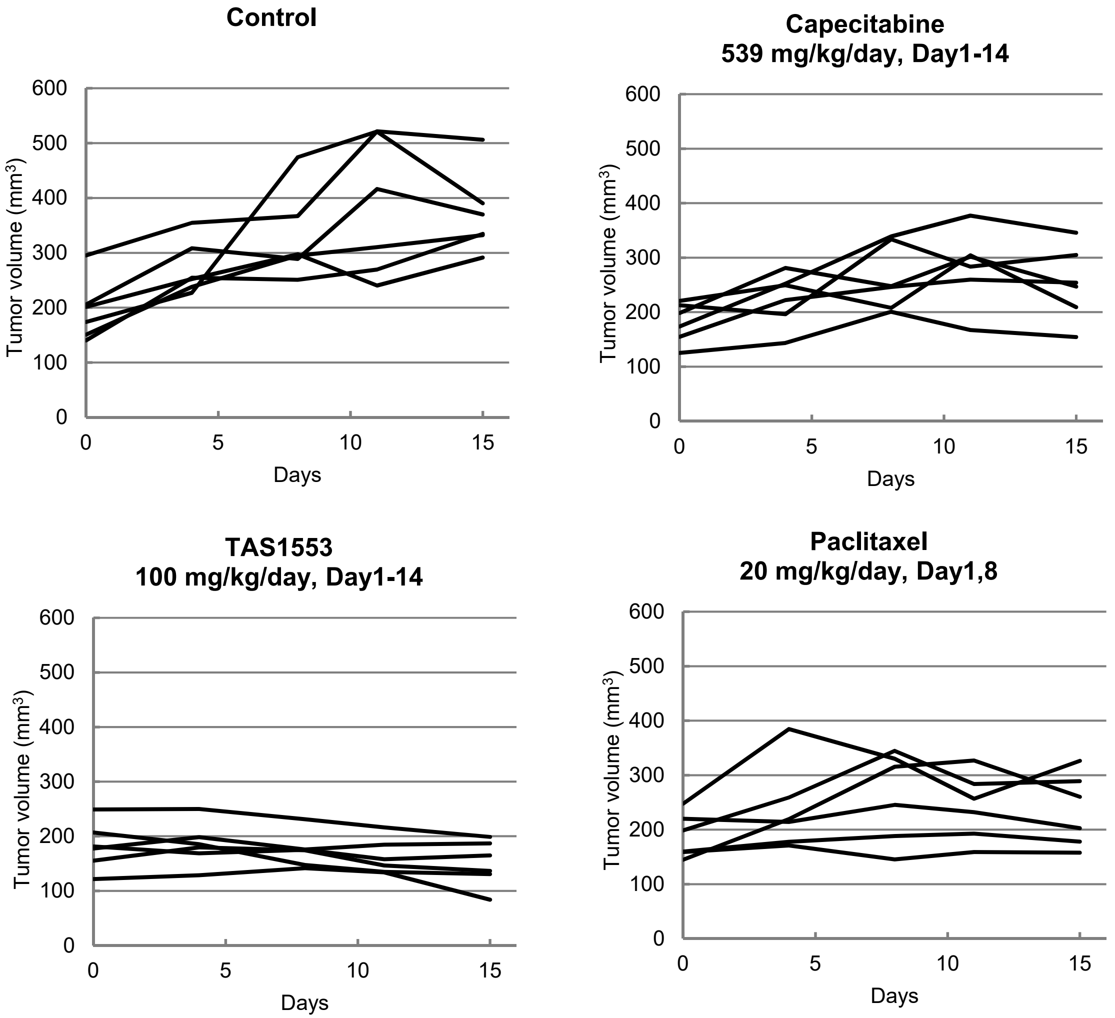

Supplementary Note 1

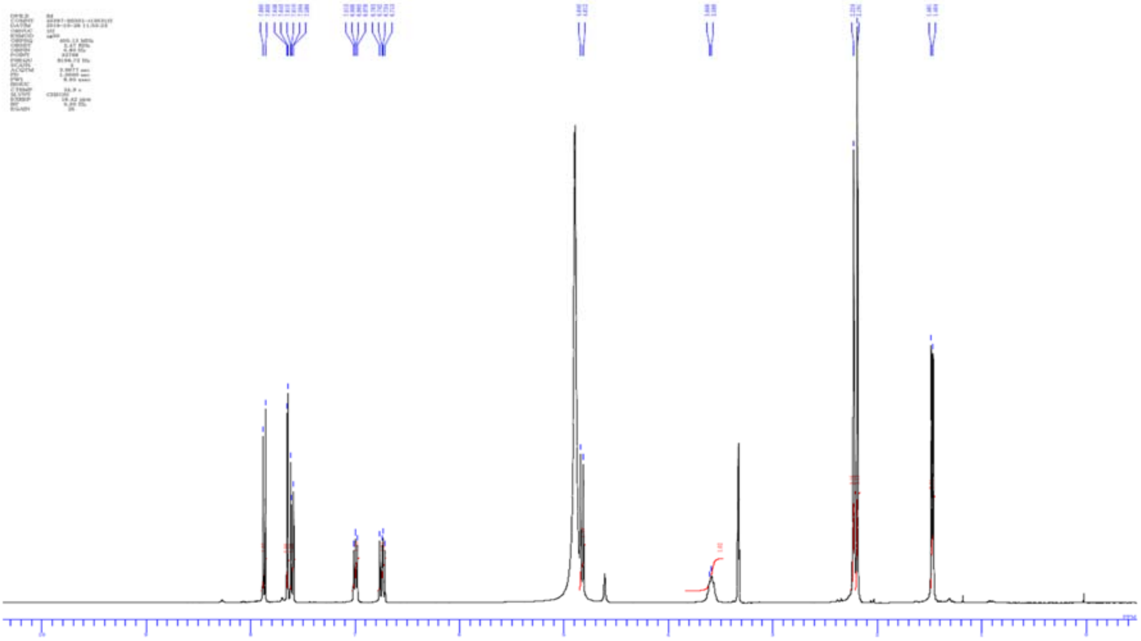

^{1}H NMR（400 MHz）spectrum of TAS1553

The ^{1}H NMR（400 MHz）spectrum of TAS1553 in CD_3OD was presented.

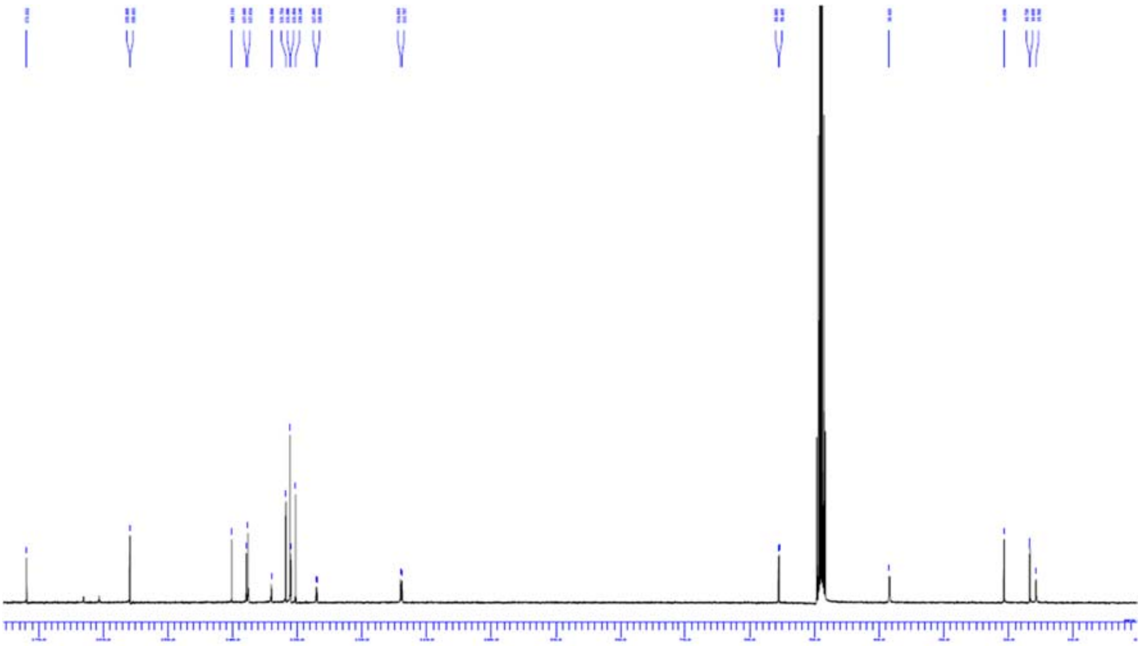

^{13}C NMR（100 MHz）spectrum of TAS1553

The ^{13}C NMR（100 MHz）spectrum of TAS1553 in CD_3OD was presented.

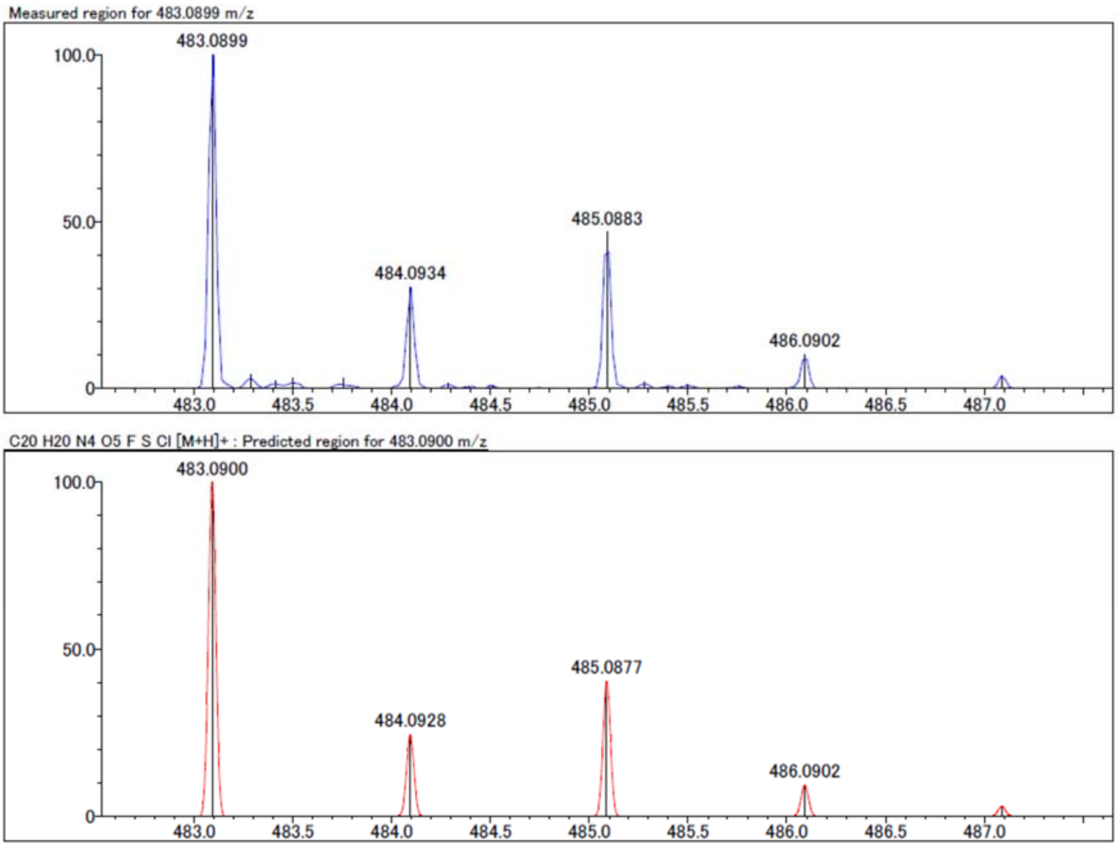

Rank	Formula (M)	Ion	Meas. m/z	Pred. m/z	Df. (ppm)	Meas. (M)
> 1	C20 H20 N4 O5 F S Cl	[M+H]+	483.0899	483.0900	−0.21	482.0826

HRESIMS of TAS1553

The result showed the HRESIMS of TAS1553

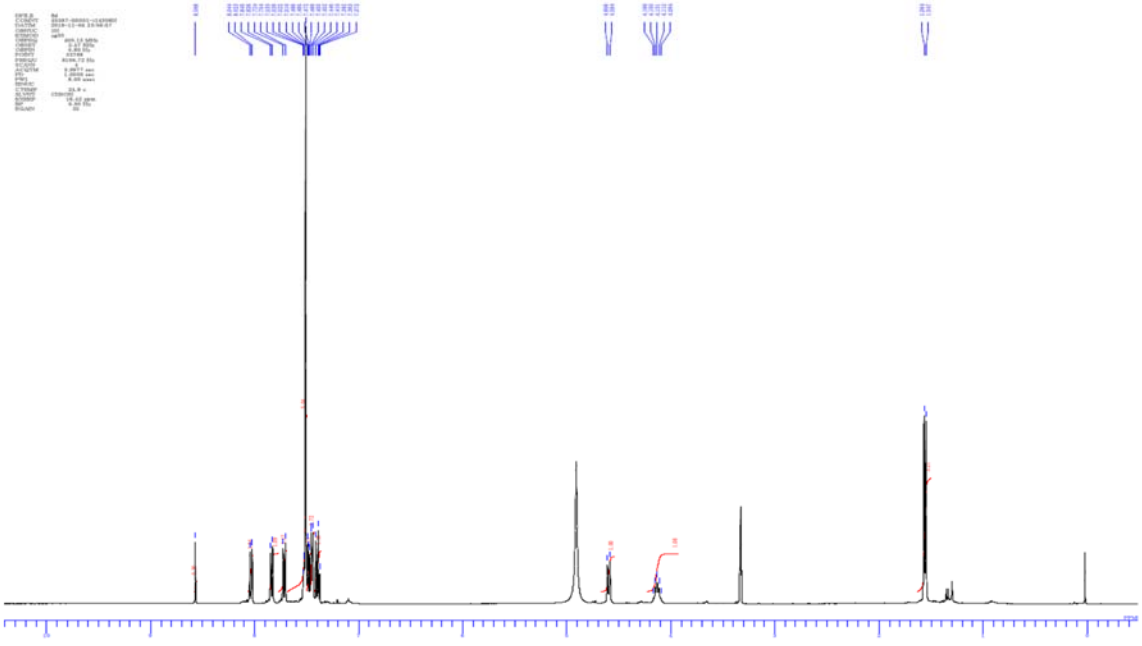

^{1}H NMR（400 MHz）spectrum of compound 1

The ^{1}H NMR（400 MHz）spectrum of compound1 in CD_3OD was presented.

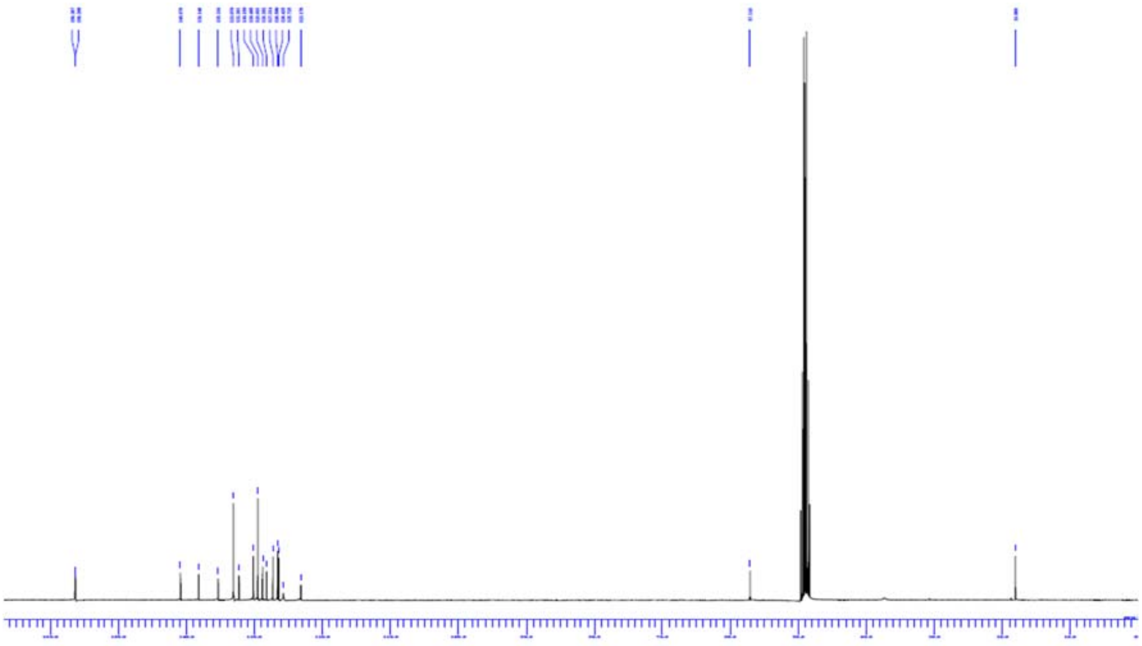

^{13}C NMR（100 MHz）spectrum of compound 1

The ^{13}C NMR（100 MHz）spectrum of compound1 in CD_3OD was presented.

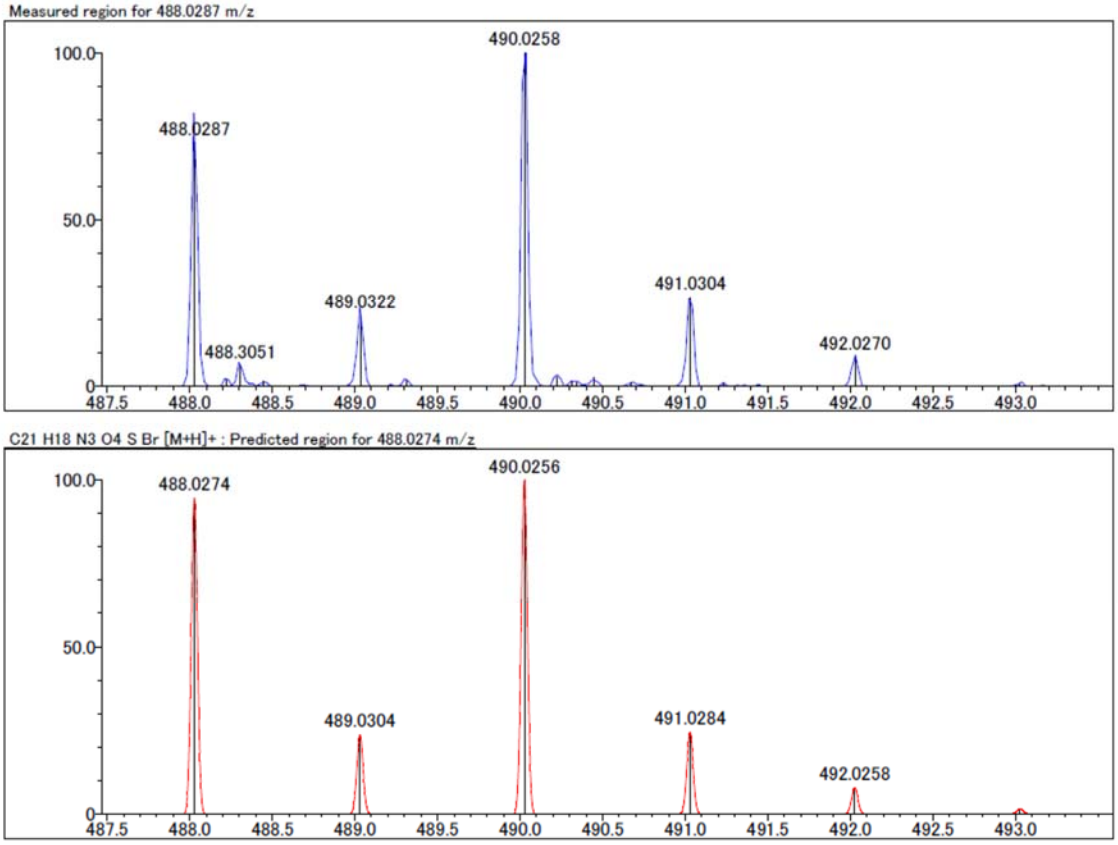

Rank	Formula (M)	Ion	Meas. m/z	Pred. m/z	Df. (ppm)	Meas. (M)
> 1	C21 H18 N3 O4 S Br	[M+H]+	488.0287	488.0274	2.66	487.0214

HRESIMS of compound 1

The result showed the HRESIMS of compound 1

Synthesis of TAS1553

TAS1553 was synthesized by a multi-step reaction. The detailed reaction schemes are as follows:

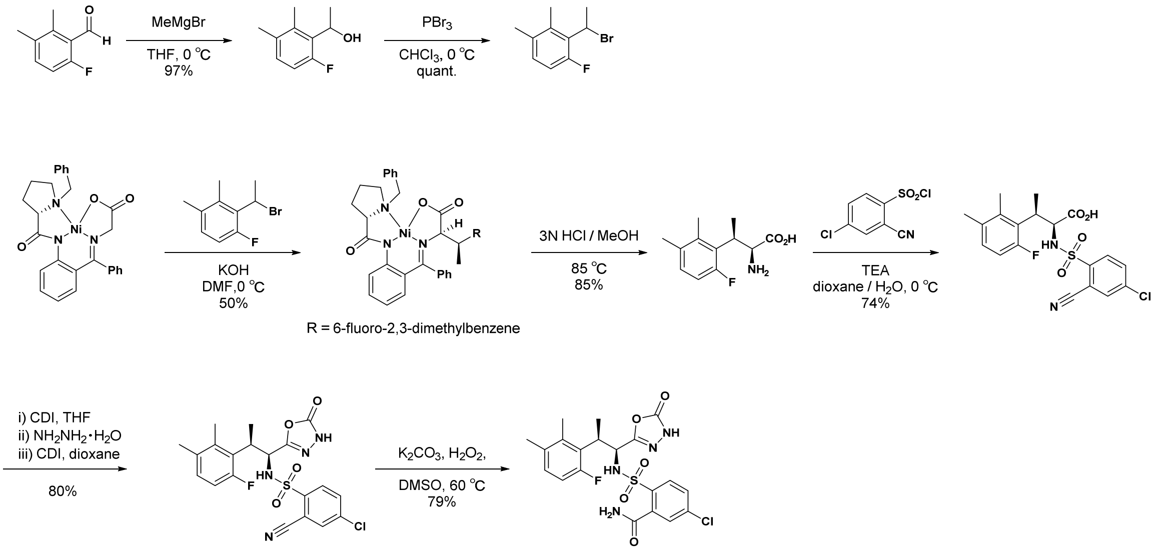

Step 1

After dropping a diethyl ether solution of methylmagnesium bromide (3.0 M, 3.2 mL, 9.6 mmol) to a THF solution (10 mL) of 6-fluoro-2,3-dimethyl-benzaldehyde (1.4g, 9.2 mmol) at 0 °C, the reaction mixture was stirred at room temperature for 30 minutes . A saturated aqueous ammonium chloride solution was added dropwise, and ethyl acetate was added, and the resultant was separated into different layers. The organic layer was successively washed with saline, and then dried over anhydrous sodium sulfate. After extracted solution was concentrated under reduced pressure, the residue was purified by silica gel column chromatography (eluent:Hexane/EtOAc = 70/30) to obtain 1.5g (98%) of 1-(6-fluoro-2,3 dimethylphenyl)ethan-1-ol as a light yellow oil.

^{1}H NMR ($CDCl_3$, 400 MHz) δ: 7.05-7.01 (1H, m), 6.84-6.79 (1H, m), 5.31-5.26 (1H, m), 2.31 (3H, s), 2.26 (3H, s), 1.61 (3H, d, J = 6.5 Hz).

Step 2

Phosphorus tribromide (14 mL, 149 mmol) was added dropwise at 0 °C to a chloroform solution (100 mL) of 1-(6-fluoro-2,3-dimethylphenyl)ethan-1-ol (12 g, 71.3 mmol) , and the reaction solution was stirred for 30 minutes at 0 °C. The reaction mixture was added to an ice-cold saturated aqueous sodium bicarbonate. After chloroform was added to the mixture, the resultant was separated into different layers, and the organic layer was successively washed with saline. The organic layer was dried over anhydrous sodium sulfate to obtain 16.5 g (quant.) by concentrating under reduced pressure.

^{1}H-NMR ($CDCl_3$, 400 MHz) δ: 7.09-7.06 (1H, m), 6.86-6.81 (1H, m), 4.23-4.12 (1H, m), 2.35 (3H, s), 2.27 (3H, s), 2.14-2.12 (3H, m).

Step 3

A DMF solution (100 mL) of 2-(1-bromoethyl)-1-fluoro-3,4-dimethylbenzene (16.0 g, 69.2 mmol) was added dropwise to a DMF solution (50 mL) of [(2*S*)-1-benzylpyrrolidine-2-carbonyl]-{2-[*N*-(carboxymethyl)-*C*-phenylcarbonimidoyl]phenyl}azanide;nickel (18.0g, 36.1 mmol), and powder potassium hydroxide (20.0 g, 356.5 mmol), and the mixture was stirred for 1 hour at 0 °C. A saturated ammonium chloride solution and ethyl acetate were added to the reaction solution, the layers were separated, and the aqueous layer was extracted twice with ethyl acetate. The combined organic layers were washed successively with water, saturated saline, dried over anhydrous sodium sulfate, and concentrated under reduced pressure. The residue was purified by silica gel column chromatography (eluent: Hexane/EtOAc = 5/95) to obtain 11.8 g (50%) as a red solid.

The obtained compound (11.8 g, 18.2 mmol) was dissolved in methanol (120 mL), then hydrochloric acid (3 M, 90 mL, 270 mmol) was added. After the mixture was stirred at 80 °C for 45 minutes, methanol was distilled off under reduced pressure, then chloroform and water were added to the residue. The aqueous layer was washed with chloroform and

concentrated under reduced pressure. The residue was purified by reverse phase silica gel column chromatography (eluent: H_2O/MeOH = 30/70) to give 3.5 g (85%) of (2*S*,3*R*)-2-amino-3-(6-fluoro-2,3-dimethylphenyl)butanoic acid as a white solid.

1H NMR (CD_3OD, 400 MHz) δ: 7.08 (dd, *J* = 8.4, 6.0 Hz, 1H), 6.82 (dd, *J* = 12.0, 8.4 Hz, 1H), 4.21-4.19 (m, 1H), 3.77-3.70 (m, 1H), 2.24 (m, 6H), 1.46-1.45 (m, 3H).

Step 4

(2*S*,3*R*)-2-amino-3-(6-fluoro-2,3-dimethylphenyl)butanoic acid (2.8 g, 12.0 mmol) was dissolved in water (100 mL) and 1,4-dioxane (100 mL). Triethylamine (5.3 mL, 38.0 mmol) was added, and the resultant was cooled to 0 °C. 4-Chloro-2-cyanobenzene-1-sulfonyl chloride (3.7 g, 16.0 mmol) was added to the reaction solution, and the mixture was stirred at the same temperature for 45 minutes. The reaction solution was added to hydrochloric acid and extracted with ethyl acetate. The organic layer was washed with saturated saline, dried over anhydrous magnesium sulfate, and concentrated under reduced pressure. The residue was purified by silica gel column chromatography (eluent: Hexane/EtOAc = 1/3, including 2% acetic acid) to give 3.9 g (74%) of (2*S*, 3*R*)-2-[(4-chloro-2-cyanophenyl)sulfonamide]-3-(6-fluoro-2,3-dimethylphenyl)butanoic acid as a white amorphous.

^{1}H NMR ($CDCl_3$ 400 MHz) δ: 7.98 (1H, d, *J* = 6.4 Hz), 7.65-7.62 (2H, m), 6.98-6.94 (1H, m), 6.68-6.63 (1H, m), 5.74 (1H, d, *J* = 10.4 Hz), 4.40-4.35 (1H, m), 3.53-3.45 (1H, m), 2.21 (3H, s), 2.18 (3H, s), 1.41-1.38 (3H, m).

Step 5

To a THF (60 mL) solution of (2*S*, 3*R*)-2-[(4-chloro-2-cyanophenyl)sulfonamide]-3-(6-fluoro-2,3-dimethylphenyl)butanoic acid (3.9 g, 9.2 mmol), CDI (2.0 g, 12.3 mmol) was added, and the reaction solution was stirred at room temperature for 1 hour. The reaction

solution was cooled to 0 ° C, hydrazine・monohydrate (1.35 mL, 27.7 mmol) was added, and the mixture was stirred at the same temperature for 20 minutes. The reaction solution was added to water and extracted with ethyl acetate. The organic layer was washed with saturated saline, dried over anhydrous sodium sulfate, and concentrated under reduced pressure. CDI (1.95 g, 12.0 mmol) was added to a 1,4-dioxane (60 mL) solution of the obtained residue, and the reaction solution was stirred at 45 ° C for 1 hour. The reaction solution was added to water and extracted with ethyl acetate. The organic layer was washed with saturated saline, dried over anhydrous sodium sulfate, and concentrated under reduced pressure. The obtained residue was purified by silica gel column chromatography (eluent: Hexane/EtOAc = 1/1) to obtain 3.4 g (77%) of 4-chloro-2-cyano-*N*-[(1*S*,2*R*)-2-(6-fluoro-2,3-dimethylphenyl)-1-(5-oxo-4,5-dihydro-1,3,4-oxadiazol-2-yl)propyl]benzenesulfonamide as a white amorphous.

^{1}H NMR (CD_3OD 400 MHz) δ: 8.03-7.99 (2H, m), 7.82 (1H, dd, *J* = 8.6, 2.2 Hz), 6.97 (1H, dd, *J* = 8.4, 5.6 Hz), 6.70 (1H, dd, *J* = 11.6, 8.4 Hz), 4.78-4.75 (1H, m), 3.68 (1H, brs), 2.20 (3H, s), 2.17 (3H, s), 1.43 (3H, d, *J* = 6.8 Hz).

Step 6

To a DMSO (70 mL) solution of 4-chloro-2-cyano-*N*-[(1*S*,2*R*)-2-(6-fluoro-2,3-dimethylphenyl)-1-(5-oxo-4,5-dihydro-1,3,4-oxadiazol-2-yl)propyl]benzenesulfonamide (3.4 g, 7.3 mmol), 30% hydrogen peroxide water (9.0 mL, 88.1 mmol) and potassium carbonate (2.2 g, 16.0 mmol) were added sequentially under an ice bath, and the reaction solution was stirred at 60 ° C for 2.5 hours. The reaction solution was slowly added to hydrochloric acid under an ice bath and then extracted with ethyl acetate. The organic layer was washed with saturated saline, dried over anhydrous sodium sulfate, and concentrated under reduced pressure. The residue was purified by

silica gel column chromatography (eluent: Hexane/EtOAc = 1/3e) to give 2.8 g (79%) of 5-chloro-2-{*N*-[(1*S*,2*R*)-2-(6-fluoro-2,3-dimethylphenyl)-1-(5-oxo-4,5-dihydro-1,3,4-oxadiazol-2-yl)propyl]sulfamoyl}benzamide (TAS1553) as a white amorphous.

$[\alpha]_D$ = -70.9 (0.5010 M in MeOH); 1H NMR (400 MHz, CD_3OD): δ 7.87 (d, *J* = 8.4 Hz, 1H), 7.65 (d, *J* = 2.0 Hz, 1H), 7.60 (dd, *J* = 8.4, 2.0 Hz, 1H), 7.00 (dd, *J* = 8.0, 6.0 Hz, 1H), 6.74 (dd, *J* = 11.6, 8.4 Hz, 1H), 4.83 (d, *J* = 11.2 Hz, 1H), 3.61-3.59 (m, 1H), 2.22 (s, 3H), 2.19 (s, 3H), 1.47 (d, *J* = 6.8 Hz, 3H); 13C NMR (100 MHz, CD_3OD): δ 171.8, 155.9, 155.9, 140.1, 137.9, 137.6, 134.0, 131.8, 131.1, 131.0, 130.2, 127.1, 126.9, 114.0, 113.8, 55.6, 55.5, 20.7; HRESIMS (m/z): calculated for $C_{20}H_{20}ClFN_4O_5S$: $[M+H]^+$ 483.0900; found : 483.0899.

Synthesis of compound 1

Compound 1 was synthesized by a multi-step reaction. The detailed reaction schemes are as follows:

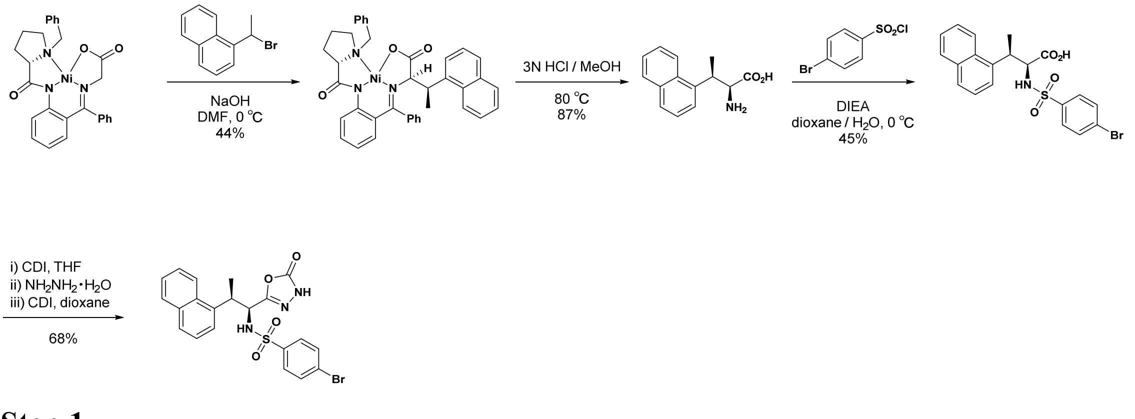

Step 1

A DMF solution (85 mL) of 1-(1-bromoethyl)naphthalene (4.2 g, 17.7 mmol) was added dropwise to a DMF solution (40 mL) of [(2*S*)-1-benzylpyrrolidine-2-carbonyl]-{2-[*N*-(carboxymethyl)-*C*-phenylcarbonimidoyl]phenyl}azanide;nickel

(4.0 g, 8.0 mmol), and powder sodium hydroxide (3.2 g, 80.3 mmol), and the mixture was stirred for 1 hour at 0 °C. A saturated ammonium chloride solution and ethyl acetate

were added to the reaction solution, the layers were separated, and the aqueous layer was extracted twice with ethyl acetate. The combined organic layers were dried over anhydrous magnesium sulfate, and concentrated under reduced pressure. The residue was purified by silica gel column chromatography (eluent: 0-80 % EtOAc in Hexane) to obtain 2.3 g (44%) as a red solid.

The obtained compound (2.3 g, 3.5 mmol) was dissolved in methanol (46 mL), then hydrochloric acid (3 M, 46 mL, 138 mmol) was added, and the mixture was stirred at 80 °C for 1 hour. After methanol was distilled off under reduced pressure, then hydrochloric acid (1 M) were added to the residue. The aqueous layer was washed with chloroform and concentrated under reduced pressure. The residue was purified by reverse phase silica gel column chromatography (eluent: 0-100 % MeOH in H_2O) to give 0.7 g (87%) of (2*S*,3*R*)-2-amino-3-(naphthalen-1-yl)butanoic acid as a white solid.

1H NMR (d6-DMSO, 400 MHz) δ: 8.18 (d, *J* = 8.8 Hz, 1H), 7.98 (d, *J* = 8.0 Hz, 1H), 7.88-7.86 (m, 1H), 7.65-7.51 (m, 4H), 4.37-4.34 (m, 1H), 3.94-3.93 (m, 1H), 1.44 (d, *J* = 7.2 Hz, 1H).

Step 2

(2*S*,3*R*)-2-amino-3-(naphalen-1-yl)butanoic acid (50.0 mg, 0.19 mmol) and 4-bromobenzenesulfonyl chloride (57.7 mg, 0.23 mmol) were dissolved in water (0.9 mL) and 1,4-dioxane (0.9 mL), the resultant was cooled to 0 °C. After *N*,*N*-diisopropylethylamine (98 µL, 0.56 mmol) was added to the reaction solution, and the mixture was stirred at the same temperature for 30 minutes. Ethyl acetate and a saturated ammonium chloride solution were added to the reaction solution, then the organic layer was washed with water, saturated saline, dried over anhydrous magnesium sulfate, and concentrated under reduced pressure. The residue was purified by silica gel column

chromatography (eluent: 0-100 % EtOAc in Hexane, including 2% acetic acid) to give 38.0 mg (45%) of (2*S*, 3*R*)-2-[(4-bromophenyl)sulfonamide]-3-(naphthalen-1-yl)butanoic acid as a colorless oil.

^{1}H NMR ($CDCl_3$, 400 MHz) δ: 8.02 (1H, d, *J* = 8.0 Hz), 7.87 (1H, d, *J* = 8.4 Hz), 7.71 (1H, d, *J* = 8.0 Hz), 7.57-7.50 (2H, m), 7.33-7.24 (3H, m), 7.10-7.02 (3H, m), 5.28 (1H, d, *J* = 8.8 Hz), 4.37-4.29 (2H, m), 1.43 (3H, d, *J* = 6.8 Hz).

Step 3

To a THF (0.85 mL) solution of (2*S*, 3*R*)-2-[(4-bromophenyl)sulfonamide]-3-(naphthalen-1-yl)butanoic acid (38.0 mg, 85 μmol), CDI (27.5 mg, 0.17 mmol) was added, and the reaction solution was stirred at room temperature for 30 minutes. Hydrazine･monohydrate (12.4 μL, 0.25 mmol) was added, and the mixture was stirred at the same temperature for 30 minutes. The reaction solution was added to water and extracted with ethyl acetate. The organic layer was washed with saturated saline, dried over anhydrous magnesium sulfate, and concentrated under reduced pressure. CDI (41.2 mg, 0.25 mmol) was added to a 1,4-dioxane (0.85 mL) solution of the obtained residue, and the reaction solution was stirred at room temperature for 1 hour. The reaction solution was added to water and extracted with ethyl acetate. The organic layer was washed with saturated saline, dried over anhydrous magnesium sulfate, and concentrated under reduced pressure. The obtained residue was purified by silica gel column chromatography (eluent: 0-100 % EtOAc in Hexane) to obtain 28.0 mg (68%) of 4-bromo-*N*-[(1*S*,2*R*)-2-(naphthalen-1-yl)-1-(5-oxo-4,5-dihydro-1,3,4-oxadiazol-2-yl)propyl]benzenesulfonamide as a white amorphous.

$[\alpha]_D$ = -69.2 (0.9300 M in MeOH); 1H NMR (400 MHz, CD_3OD): δ 8.03 (d, *J* = 8.4 Hz, 1H), 7.84 (d, *J* = 7.6 Hz, 1H), 7.71 (d, *J* = 8.0 Hz, 1H), 7.53-7.52 (m, 5H), 7.51-7.47 (m,

1H), 7.47-7.44 (m, 1H), 7.10-7.37 (m, 1H), 4.60 (d, *J* = 8.0 Hz, 1H), 4.17-4.09 (m, 1H), 1.56 (d, *J* = 6.8 Hz, 3H); 13C NMR (100 MHz, CD_3OD): δ 156.4, 156.3, 140.9, 138.2, 135.3, 133.1, 132.3, 130.2, 129.5, 128.8, 128.2, 127.3, 126.6, 126.4, 125.7, 123.1, 57.1, 18.1； HRESIMS (m/z): calculated for $C_{21}H_{18}BrN_3O_4S$: $[M+H]^+$ 488.0274; found, 488.0287.
