# Supplementary material for: TAS1553, a small molecule subunit interaction inhibitor of ribonucleotide reductase, exhibits antitumor activity by causing DNA replication stress
Source: Commun Biol. 2022 Jun 9;5:571. doi: 10.1038/s42003-022-03516-4 (PMC9184620; doi:10.1038/s42003-022-03516-4)
Supplement: Supplementary file 2 — Reporting Summary [file 42003_2022_3516_MOESM2_ESM.pdf]

nature research

Corresponding author(s): Hiroyuki Ueno

Last updated by author(s): 04/07/2022

Reporting Summary

Nature Research wishes to improve the reproducibility of the work that we publish. This form provides structure for consistency and transparency in reporting. For further information on Nature Research policies, see our Editorial Policies and the Editorial Policy Checklist.

Statistics

For all statistical analyses, confirm that the following items are present in the figure legend, table legend, main text, or Methods section.

n/a	Confirmed	
☐	☒	The exact sample size (n) for each experimental group/condition, given as a discrete number and unit of measurement
☐	☒	A statement on whether measurements were taken from distinct samples or whether the same sample was measured repeatedly
☐	☒	The statistical test(s) used AND whether they are one- or two-sided<br>*Only common tests should be described solely by name; describe more complex techniques in the Methods section.*
☒	☐	A description of all covariates tested
☐	☒	A description of any assumptions or corrections, such as tests of normality and adjustment for multiple comparisons
☐	☒	A full description of the statistical parameters including central tendency (e.g. means) or other basic estimates (e.g. regression coefficient) AND variation (e.g. standard deviation) or associated estimates of uncertainty (e.g. confidence intervals)
☒	☐	For null hypothesis testing, the test statistic (e.g. F, t, r) with confidence intervals, effect sizes, degrees of freedom and P value noted<br>*Give P values as exact values whenever suitable.*
☒	☐	For Bayesian analysis, information on the choice of priors and Markov chain Monte Carlo settings
☒	☐	For hierarchical and complex designs, identification of the appropriate level for tests and full reporting of outcomes
☒	☐	Estimates of effect sizes (e.g. Cohen's d, Pearson's r), indicating how they were calculated

Our web collection on statistics for biologists contains articles on many of the points above.

Software and code

Policy information about availability of computer code

Data collection	The surface plasmon resonance was measured with Biacore T200 (Evaluation Software, version 3). dCDP was quantified by a prominence HPLC system (LabSolutions, ver 5.54 SP1). LC-MSMS analysis was performed with a prominence HPLC system connected to a LCMS-8040 tandem mass spectrometer with ESI probe (LabSolutions, ver 5.91).
Data analysis	IC50 values were calculated using the SAS software package (ver 9.2) in EXSUS (ver 8.0.0). The program iMOSFLM from the CCP4 suite was used to process the data. The structure was refined using REFMAC5. Calculation of affinity (Kd) value was performed with Biacore T200 Evaluation Software (version 3). dCDP for RNR assay and intracellular metabolites were measured with LabSolutions (ver 5.54 SP1) and LabSolutions (ver 5.91), respectively. Correlation analysis was performed using Pearson's correlation coefficient in JMP 13 software (ver 13.2.0). Dunnett test and Log-Lank test using the SAS software package (ver 9.2) in EXSUS (ver 8.0.0) was used to compare the tumor volume and survival time, respectively.

For manuscripts utilizing custom algorithms or software that are central to the research but not yet described in published literature, software must be made available to editors and reviewers. We strongly encourage code deposition in a community repository (e.g. GitHub). See the Nature Research guidelines for submitting code & software for further information.

Data

Policy information about availability of data

All manuscripts must include a data availability statement. This statement should provide the following information, where applicable:

- Accession codes, unique identifiers, or web links for publicly available datasets
- A list of figures that have associated raw data
- A description of any restrictions on data availability

Cocrystal structure : 6L3R and 2ZLF (PDB ID)
CCLE : http://www.broadinstitute.org/ccle/home

Field-specific reporting

Please select the one below that is the best fit for your research. If you are not sure, read the appropriate sections before making your selection.

☒ Life sciences ☐ Behavioural & social sciences ☐ Ecological, evolutionary & environmental sciences

For a reference copy of the document with all sections, see nature.com/documents/nr-reporting-summary-flat.pdf

Life sciences study design

All studies must disclose on these points even when the disclosure is negative.

Sample size	No sample size calculation was performed for in vitro studies. n=3/group and n=5-10/group were used for in vivo PD studies and efficacy studies, respectively, based on introductory investigation.
Data exclusions	No data were excluded.
Replication	In vitro experiments were repeated as indicated in figure legend. Immunoblot experiments and immunofluorescence experiments were repeated independently at least once. All in vivo studies were performed once.
Randomization	Sample randomization is not relevant to the in vitro studies. For in vivo studies, animals were randomized to the test groups by using the MiSTAT Grouping Program.
Blinding	Blinding was not performed.

Reporting for specific materials, systems and methods

We require information from authors about some types of materials, experimental systems and methods used in many studies. Here, indicate whether each material, system or method listed is relevant to your study. If you are not sure if a list item applies to your research, read the appropriate section before selecting a response.

Materials & experimental systems

n/a	Involved in the study	
☐	☒	Antibodies
☐	☒	Eukaryotic cell lines
☒	☐	Palaeontology and archaeology
☐	☒	Animals and other organisms
☒	☐	Human research participants
☒	☐	Clinical data
☒	☐	Dual use research of concern

Methods

n/a	Involved in the study	
☒	☐	ChIP-seq
☒	☐	Flow cytometry
☒	☐	MRI-based neuroimaging

Antibodies

Antibodies used	Anti-Chk1 antibody (#2360), anti-Phospho-Chk1 (Ser345) antibody (#2348), anti-Cleaved PARP antibody (#5625), anti-Cleaved Caspase-3 antibody (#9661), anti-β-Actin antibody (#4967), HRP-conjugated anti-rabbit IgG antibody (#7074) and HRP-conjugated anti-mouse IgG antibody (#7076) were obtained from Cell Signaling Technology, Inc. Anti-phospho-RPA2 (Thr21) antibody (#ab61065) and anti-RRM1 antibody (#ab137114) were obtained from Abcam plc. Anti-SLFN11 antibody (#sc515071) and anti-RRM2 antibody (#sc10844) were obtained from Santa Cruz Biotechnology, Inc. Anti-RPA2 antibody (#NA19L), anti-phospho-RPA2 (Ser4/Ser8) antibody (#A300-245A) and anti-γH2AX antibody (#613402) were obtained from Merck KGaA, Bethyl Laboratories, Inc., and BioLegend, Inc., respectively.
Validation	All antibodies were validated by the manufacturers.

Eukaryotic cell lines

Policy information about cell lines

Cell line source(s)	The Ca9-22, HEL, NUGC-3, and RPMI8226 cell lines were obtained from the Japanese Collection of Research Bioresources. The 786-O, CFPAC-1, DU145, HCC1599, HCC1806, HCC38, HCT116, HL-60, K-562, MSTO-211H, MV-4-11, NCI-H460, NCI-H2170, and THP-1 cell lines were obtained from the ATCC. The A2780, COLO 792, and DOK cell lines were obtained from the European Collection of Authenticated Cell Cultures. The A549, A673, BHL-89, and MCF-7 cell lines were obtained from DS Pharma Biomedical Co., Ltd.
Authentication	Short-tandem repeat-based DNA profiling was used to reauthenticate cell lines.
Mycoplasma contamination	All cell lines were confirmed to be negative for mycoplasma contamination.
Commonly misidentified lines (See ICLAC register)	No commonly misidentified cell lines were used.

Animals and other organisms

Policy information about studies involving animals; ARRIVE guidelines recommended for reporting animal research

Laboratory animals	F344/NJcl-rnu/rnu rats : female, 4 weeks, CLEA Japan<br>BALB/cAJcl-nu/nu mice: male, 5 weeks, CLEA Japan<br>B6N-Tyrc-Brd/BrdCrCrl mice : male, 5 weeks, Charles River Laboratories
Wild animals	Wild animals were not used.
Field-collected samples	Field-collected samples were not used.
Ethics oversight	Animal protocols were approved according to regional Institutional Animal Care and Use Committees

Note that full information on the approval of the study protocol must also be provided in the manuscript.
